# Bovine tuberculosis control in Fiji: Retrospective study findings for 2015 to 2020

**DOI:** 10.3389/fvets.2022.972120

**Published:** 2022-09-30

**Authors:** Anabel Argelis Garcia, Elva Borja, Aoife Reid, Vijendra Samy, Shivani Singh, Richard J. Whittington, Jenny-Ann L. M. L. Toribio

**Affiliations:** ^1^Sydney School of Veterinary Science, Faculty of Science, The University of Sydney, Camden, NSW, Australia; ^2^Ministry of Agriculture, Koronivia Research Station, Nausori, Fiji

**Keywords:** bovine tuberculosis, disease control, cattle, BTEC, Fiji

## Abstract

Control of bovine tuberculosis (bTB) is a priority for animal health, biosecurity, and human health authorities in Fiji as evident from the long-term funding of the Bovine Brucellosis and Tuberculosis Eradication and Control program (BTEC) and notable improvements to the program described in this paper. To evaluate the performance of the Fiji BTEC program from 2015 to 2020, all available bTB data for cattle were analyzed. Data sources included BTEC bTB testing records, abattoir records and laboratory records. We integrated all information to quantify the bTB tests applied, bTB positive farms and animals, meat inspection and laboratory findings. Test coverage was highest among dairy cattle in Central Division (~73%), where bTB was highly prevalent with 7.8% of dairy cattle and 61.7% of dairy farms found to be positive between 2015 and 2020. There was no visible downward trend in the apparent prevalence of bTB over the 6-year period. During 2019 and 2020, only 21.3% (51/239) of the tested dairy farms maintained their clear status, another 8.4% (20/239) reverted to infected status after 1 year or more of being bTB clear, and most farms remained infected during these 2 years. Factors observed to be contributing to this situation were persistent infections, related in part to the significant number of untested animals, uncontrolled animal movements, and larger farm size. Similar to other developing countries, bTB remains a serious concern and further strengthening of the program targeting the main contributors to bTB persistence, along with maintenance of a comprehensive reporting and traceability system, industry awareness and government support are needed. Control of bTB in Fiji is a long-term objective that must have multiple stakeholder engagement and regular review to measure success.

## Introduction

Bovine Tuberculosis (bTB) is primarily caused by *Mycobacterium bovis* and is a chronic disease that constitutes a significant economic burden to cattle production industries (1, 2). bTB is a geographically widespread infectious disease (https://www.cabi.org/isc/datasheet/91739). It is transmitted between cattle and people, and cattle and wildlife making control a substantial challenge for public health and animal health systems (3, 4). Programs to control bTB in cattle are generally based on test and cull, implementation of hygienic practices, certification of bTB-free farms and slaughter-based surveillance; the components vary according to the goals of the control program in a particular country and the level of disease therein (5). In high-income countries where control programs have been sustained for lengthy periods, progressive reduction in distribution and prevalence has often been achieved. However, the eradication of bTB from cattle populations (extinction of *M. bovis*) has been achieved by few countries, notably Australia (6). In terms of legal freedom, several countries and regions across Northern Europe, Asia, and America are declared free of bTB based on bTB testing over a period of 3 years or more with no evidence of bTB in at least 99.8% of farms representing 99.9% of the total cattle population (7–9). *M. bovis* in wildlife reservoirs is described to be the main reason for the persistence of the disease hampering biological freedom of bTB in high-income countries such as New Zealand, UK, and USA (6, 7, 10, 11). In most middle to low-income countries where bTB is endemic, even planning a disease control program can be a challenge due to insufficient data collection to determine the epidemiological situation, which is essential information to effectively allocate scarce resources for bTB control (12–14).

Globally there is a renewed focus on control of bTB because it is linked to the World Health Organization (WHO) strategy to end the human tuberculosis (TB) epidemic with targets for large reductions in TB incidence, TB-related deaths and household costs by 2030. People at risk of zoonotic TB are often members of neglected populations deserving greater attention. Thus, a Roadmap for Zoonotic TB was endorsed by WHO's Strategic and Technical Advisory Group for TB in 2016 (15).

In Fiji, the need to consider zoonotic TB in the national TB program is reflected in the designation of bTB as a One Health challenge by the Fiji Ministry of Agriculture (MOA) and the Ministry of Health and Medical Services (MHMS) (16). The recognition of the detrimental impacts for animal health and human health has motivated research, collaboration and information sharing by the two ministries.

In fact, control of bTB has been a priority for the Government of Fiji for more than two decades, evidenced by the consistent annual funding of the Bovine Brucellosis and Tuberculosis Eradication and Control program (BTEC). Cattle are highly valued in this Pacific-island nation, which despite limited farmland (10.7% of total aggregate land area) raises a total of 119,691 cattle (70,041 beef cattle and 49,650 dairy cattle) under extensive grazing management according to the 2020 Fiji Agricultural Census (2020 FAC) (17). This is one head of cattle for every eight people in Fiji based on the 2019 human population total of 906,784 (17, 18). The dairy industry is particularly significant for the country, providing 79.5% (1,696,695 liters of fresh milk) of total volume for sale according to a 3-month analysis by the 2020 FAC (17). Whilst the BTEC program involves conduct of tests for brucellosis and for tuberculosis during a farm visit, this study focused solely on bTB data, in part because there were no positive brucellosis test results during the 6-year study period. This study is one example of the work undertaken, and it addresses a priority area of the Roadmap for Zoonotic TB to improve the scientific evidence base: surveillance and reporting of better quality data on bTB in livestock (15). Zoonotic aspects of bTB in Fiji will be reported separately.

The key activities of the Fiji BTEC program over time have been mandatory on-farm bTB testing using the single intradermal test (SID) with unique individual identification of tested animals, data recording and analysis; removal of infected cattle from farms with compensation; laboratory and abattoir surveillance; and cattle movement control to reduce and prevent infection spread from infected farms. However, a retrospective investigation of BTEC bTB data drawn from hard-copy records for the 16 years from 1999 to 2014 demonstrated that despite sustained funding of the BTEC program, disease reduction and containment was not being achieved (19). This finding provided the needed justification for the MOA to implement changes in 2014 in the reading of the SID to improve the sensitivity of detection of infected animals in the field. In 2016, the Biosecurity Authority of Fiji also implemented movement restrictions through the Biosecurity Emergency Area Declarations that states that any movement of cattle and calves within Fiji is strictly prohibited unless BAF provides prior authorization for the movement. Farmers must complete the required cattle movement application form and submit it to a BAF office at least 7 days before the movement date. A farm's bTB status, which is determined by the MOA's BTEC team based on BTEC on-farm testing results, must be a “Clear Status” for BAF to issue the movement permit.

These changes had some economic consequences for the dairy industry, such as a reduction of herd size on some dairy farms due to culling of bTB positive animals (also called reactors) and led to concern about the limited replacement stock being available in Fiji. Furthermore, as the prevalence of animals with generalized and gross bTB lesions in infected farms decreased, a greater proportion of culled reactors had no-visible lesions (NVL) on post-mortem inspection at the abattoir. All this raised questions about the sustainability of using the SID testing protocol (20). As a response to farmer concerns, the first BTEC stakeholder's forum was organized by the MOA in 2017 to encourage participation of the industry in making decisions for implementation of the BTEC program. One of the important outcomes of the 2017 forum was the endorsement of a new BTEC Strategy by industry stakeholders in 2018. This strategy included enhanced criteria for the identification of infected cattle farms based on the SID test, immediate removal of infected cattle with an improved compensation scheme, upskilling of meat inspectors, and strengthened implementation of restrictions on cattle movements.

Under the new strategy, BTEC information was intentionally incorporated into the design of a new cloud data platform *Bovibase*, to record farm details on location, production, livestock, farm testing and cattle movement, with the intention of data sharing using *Bovibase* as a national recording system for the cattle industry. *Bovibase* was launched in 2019 during a 2^*nd*^ BTEC forum, enabling the recording of on-farm testing, infectious status, and meat inspection results for bTB reactor animals. Functionally, BTEC staff were able to access information about farms that were due for bTB testing, thereby aligning testing dates and reading dates, and prioritizing infected farms. Furthermore, data for animals with an incomplete bTB test and missing animals were recorded.

This paper presents a detailed collation and analysis of BTEC bTB records from 2015–2020, providing estimates of the coverage of the BTEC program and of the level of bTB infection in cattle herds in Fiji. Furthermore, it provides evidence of factors that are contributing to the transmission and maintenance of bTB in dairy cattle and guidance for updating the BTEC strategy based on the progress that has been made to date. These findings extend beyond the previous retrospective study for 1999–2014 and demonstrate the need for continued investment and collaboration between the government and the cattle industry in Fiji to achieve sustained control of bTB.

## Materials and methods

A retrospective study was conducted using bTB test data of the Fiji BTEC program from 2015 to 2020. Approval from the Ministry of Agriculture (MOA) to conduct data analysis was received in March 2020. Data preparation and analysis were conducted from November 2020 to October 2021. The diagnostic test used throughout the 6-year period was the SID test using purified protein derivative tuberculin antigen for *M. bovis* (PPD-B) injected intradermally into the caudal skin fold of the tail (CFT), with the result read 3 days after administration. Over the 6-years, it was deemed to be a positive test if the animal had developed a wheal of any size or redness at the injection site, following the OIE recommendation for detection of reactors in known infected farms (2). This test reading criteria, based on the assumption that all cattle in Fiji are potentially bTB infected, was implemented from 2014 regardless of whether a farm was previously identified as disease-clear or infected status (21).

The determination of clear or infected status was based on the following criteria: farms with reactors determined by the SID were classified as “Infected”; and for an infected farm to be determined as “Clear” from bTB, it was necessary for it to have 3 consecutive negative SID tests at a minimum of 3-month intervals to obtain “Restricted', “Provisionally clear,” and then “Clear” statuses, respectively. Upgrading to a Clear status also relied on the compliance of the farmer to present all cattle older than 3 months of age for complete bTB testing (i.e., presented for tuberculin injection and then again for reading of the result) (21).

### Data sources, bTB test data entry and verification

**Figure 1:**
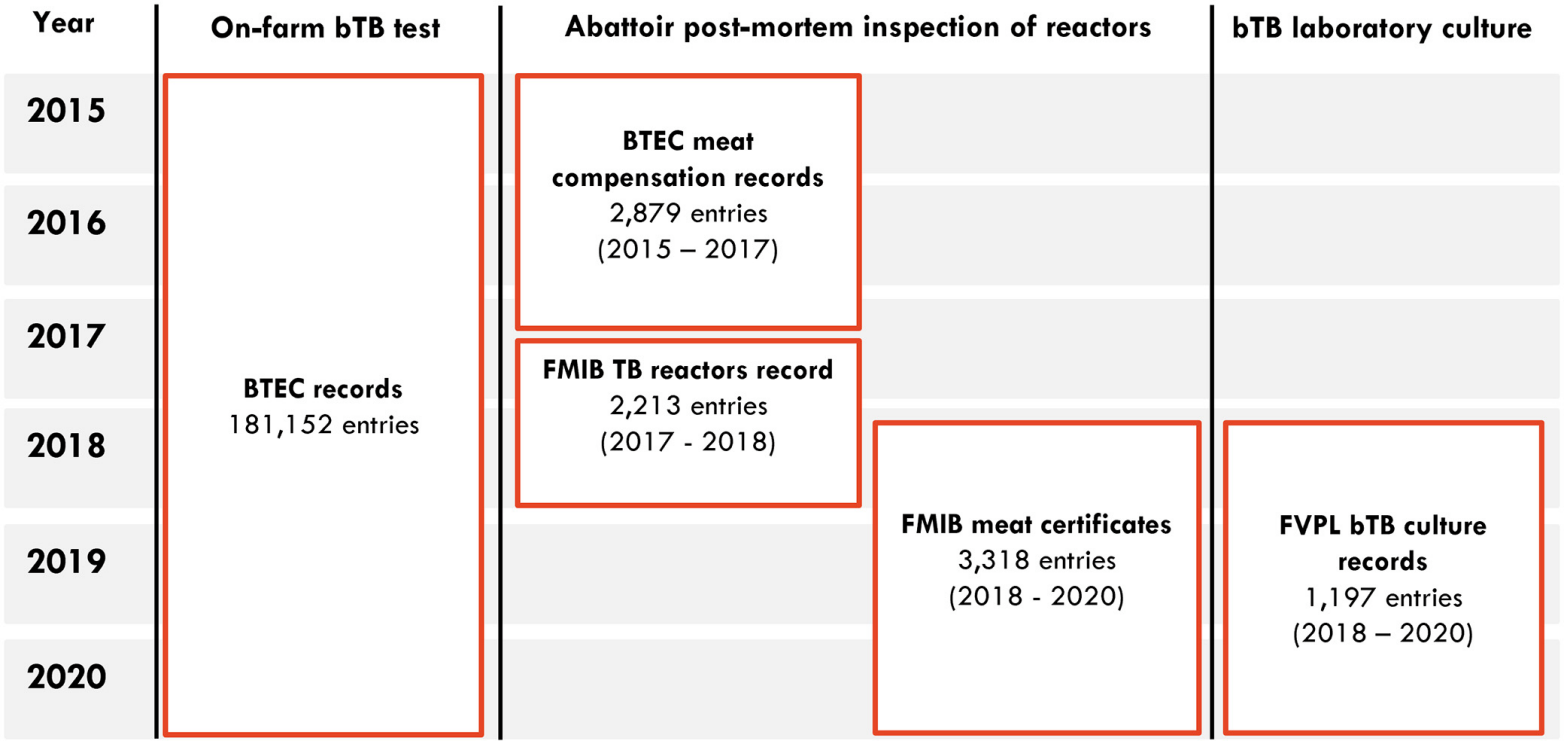
Fiji BTEC data sources used in this study from 2015–2020.

Several types of data were collated and used in this study (Figure 1). Due to weak record management systems and the collation of data from multiple sources, the processes for data collation, cleaning and verification were complex and time consuming, with this work extending over 11 months.

Soft copies of BTEC bTB test data from 2015 to 2017 encoded in Microsoft Excel were provided by the MOA. For 2018, a soft copy of some data was provided, and the remainder was subsequently encoded in MS Excel by BTEC personnel. The data from 2015 to 2018 were systematically checked using MS Excel (Microsoft^®^ Excel^®^ for Microsoft 365 MSO (16.0.14326.20908)) and R (version 4.0.4) to remove duplicate records and correct or delete errors after comparison against hard copies. Verification was performed by cross-checking against the unique identity farm (see below).

For 2018, a marked difference in totals was identified between verified bTB testing data, the BTEC annual report and the abattoir reports of bTB positive animals detected on farm and subsequently sent to slaughter. Consequently, the total time period for data verification was lengthy (from January to September 2021) and included an additional search of BTEC hard copy records and of soft copy files to exhaust all possible data sources, but no other files were found. Therefore, it was assumed that some 2018 test data records were permanently missing.

For 2019 and 2020, complete data were downloaded from the BTEC program section of the cloud data platform *Bovibase*. The data provided by *Bovibase* were verified by cross-checking against the hard copies and confirmed to be correct.

For farms visited only during 2015 to 2018, farm identification was based on unique farm names linked to the MOA dairy registration number or village, settlement, or town supply descriptions. For farms visited during 2019–2020, those data consistently contained a unique farm identification code (*Bovibase* ID) for each farm visited, and for farms that had also been visited previously during 2015 to 2018, the *Bovibase* ID was added to the earlier records.

Dairy farm identification was verified by matching the BTEC farm registration number and the farm name to the MOA dairy farm registration list; as each farm was required to register with the MOA on an annual basis and a new, different registration number was allocated each year, this prevented the analysis of data for a specific farm across all the years of the study. Following introduction of *Bovibase*, each dairy farm visited by BTEC in 2019 received a new, unique *Bovibase* ID that was maintained from 2019 onwards.

Validation of beef farms was based on familiarity of BTEC staff with farmers and farm operations as the MOA had no formal registration system for beef farms. From 2019, a unique *Bovibase* ID was allocated to each beef farm.

A list provided by the MOA Economic Planning and Statistics Division (EP&S) was used to validate location details recorded for each farm.

In the results section, the term “Unique identity farm” was used to indicate the count of unique *Bovibas*e ID or farm name. There were 1,280 unique identity farms (dairy, beef and holding facilities).

All the data compiled and used for further analysis are described in Supplementary Table 1.

### National cattle population data

**Figure 2:**
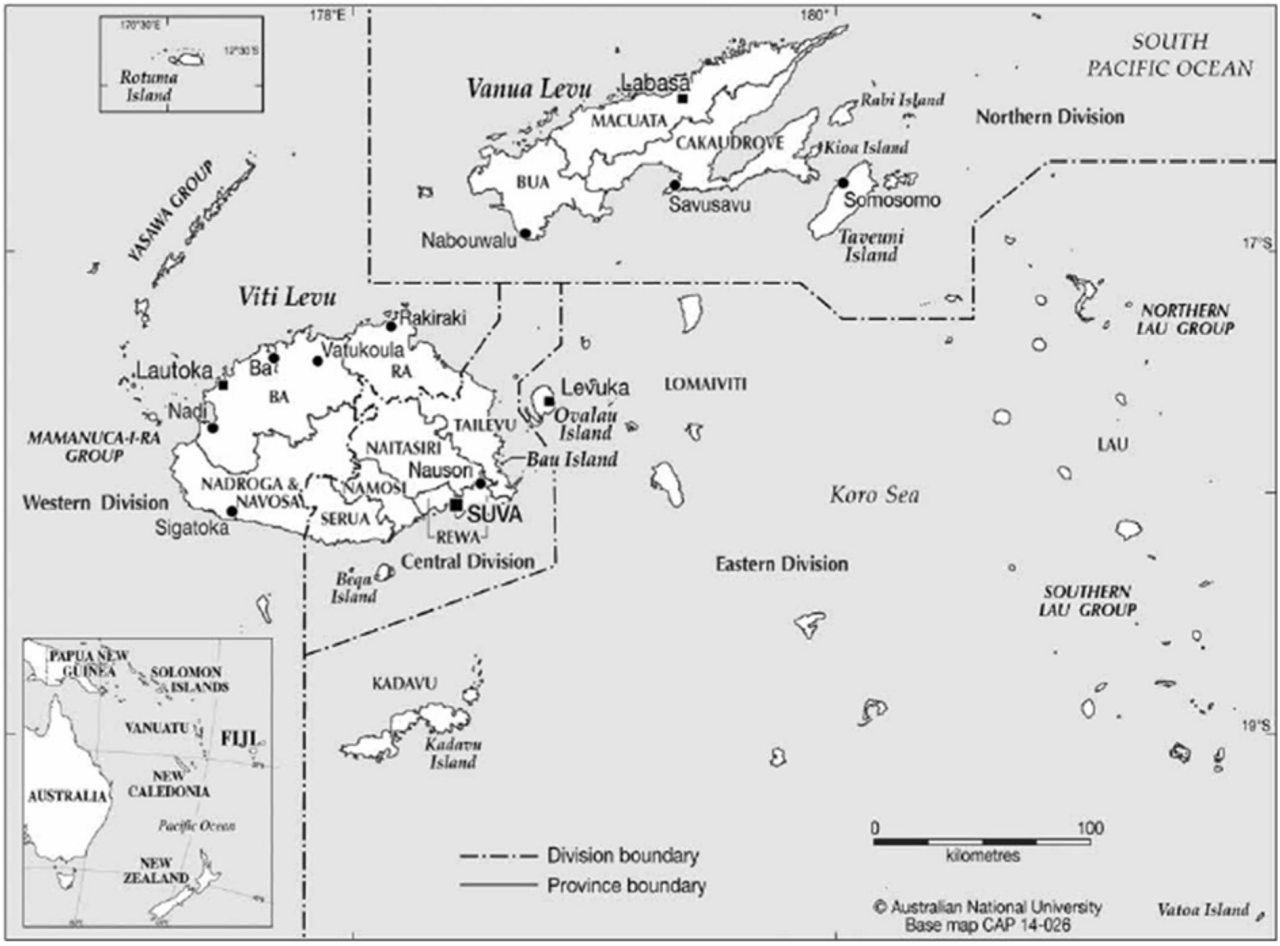
Map of Fiji showing Division boundaries. Maps Online, CartoGIS Services, ANU College of Asia and the Pacific, The Australian National University.

As a national program, BTEC is responsible for investigation of cattle in Central, Western, Northern and Eastern divisions of Fiji (Figure 2). BTEC program population coverage of testing was estimated using the national cattle population figures from the 2020 Fiji Agricultural Census (15) as the denominator.

### Identification and classification of farms

Farms of individual farmers, school farms, villages/settlements, government stations, cattle traders and impoundments were classified by type of operation (dairy, beef, holding facility). Dairy refers to an establishment that produced milk for a bulk supplier, town supply or for personal consumption, and/or traded dairy cattle. Beef refers to an establishment that only raised and/or traded cattle for meat production. Holding facilities refers to a site designated for holding impounded straying cattle with and without ear tags for short periods of time, (commonly called “pounds” or “impoundments)”.

Dairy farms that had registered and paid a license fee to the MOA to officially sell milk to Fiji Dairy Limited (FDL) are referred as licensed dairy farms. These licensed farms must comply with additional regulations related to hygiene and sanitary standards to be authorized to sell milk. BTEC prioritizes the testing of these farms under the BTEC program with the aim to improve national dairy production and the safety of their dairy products. Among these licensed farms, the commercial farms that usually have higher milk production are called bulk suppliers, and Fiji Dairy (PTE) Limited collects the milk from the farm using the company-owned bulk tankers to transport it to the milk processing factory at Nabua, Fiji. For the smaller licensed dairy farms, the collection of milk from the farm in aluminum milk cans is coordinated by the Fiji Cooperative Dairy Company Limited (FCDCL) which operates chilling centers where the milk is stored for cooperative members until collection by Fiji Dairy Limited.

Farms were classified based on herd size as commercial, semi-commercial or subsistence. A commercial farm was a farm with more than 40 cattle, a semi-commercial farm was a farm with 16 to 40 cattle and a subsistence farm was a farm with 1 to 15 cattle. Herd size was based on the median number of cattle bTB tested across the visits to a farm. If a farm was tested during 2015 to 2018 only, the median was calculated based on the total cattle tested per test visit during that period. If a farm was tested during 2019 to 2020, when untested animals were not counted, then the median was calculated based on the total cattle tested per test visit over these 2 years.

### Individual animal data

Individual animals were identified by metal ear tags each with a unique number provided by BTEC. In the event that a tag was lost a new tag was provided by the BTEC Program. In the results section, the term “Unique identity animal” was used to indicate the unique metal ear tag animal identification listed on BTEC records.

#### On-farm testing

From 2015 to 2017, bTB SID tests were performed on animals older than 6 months and from 2018 onwards the tests were performed on animals older than 3 months as part of the enhancement of the standard operating procedures (SOP). For 2015 to 2018, it was assumed that all bTB SID test administered on Day 1 were read 3 days after the day of PPD-B tuberculin administration (Day 4). From 2019 the actual reading date was recorded in *Bovibase*, as well as the unique identity animal number of the animals that were not presented on the reading date (recorded as “Not Presented” or “NPD4”). The unique identity animal for animals that were presented by the farmers on Day 4 but that were not presented for testing on Day 1 were also recorded from 2019 (recorded as “Not Tested)”. The term “Untested animal” was used to describe both “Not Presented” and “Not Tested” animals.

Furthermore, during 2019 and 2020, *Bovibase* was enhanced to classify unique identity animal numbers into a category called “missing animals” when not presented on Day 1 and Day 4 during a BTEC visit, without record of death, slaughter, or sale. When an animal was recorded as missing during 3 consecutive BTEC tests on a farm it was subsequently removed from the farm total stock.

The dataset compiled from 2015 to 2018 included: date of test, animal identification number (metal tag), animal type (milking cow, bull, bull calf, cow, dry cow, heifer, heifer calf, steer), animal gender (female or male), farm registration number, farm name, farm location (division, province, village/settlement, locality) and bTB SID results (Positive, Negative). From 2019 to 2020, the dataset also included: farm *Bovibase* ID, animals not presented on Day 4 for bTB test reading (NPD4), and animals not tested (Not Tested), date of reading, batch number and next test date.

#### Abattoir post-mortem inspection of reactors

Complete data for the post-mortem inspection of all reactors, issued by the meat inspectors of the MOA regulatory unit, were available only for the FMIB Nasinu abattoir. However, these records were not available for 2015 and 2017, thus Meat Compensation records issued by BTEC were used instead (Figure 1). These records included: unique identity animal number, unique identity farm, farm name, farm location, animal color, gender, stock movement number, type of lesion detected, payment rate ($), compensation percentage (%), total compensation ($) and lesion weight (kg).

From 2017 to 2020, records of the reactors' post-mortem inspection obtained from the FMIB Nasinu abattoir were, the “TB reactors record” from 2017 to 2018, and the “Meat Certificates” from 2018 to 2020 (Figure 1). TB reactors record and Meat certificates datasets included: animal identification number, farm registration number, farm name, farm location, animal type, animal gender, stock movement number, date of slaughter and type of lesion detected. Meat certificates provided additional data such as the meat certificate number and lesion weight for inspections done before 2019 and dressed carcass weight for inspections done from 2019.

There were two major categories of outcome for bTB reactors recorded by meat inspectors at the abattoir: no visible lesions of bTB (NVL) or visible lesions of bTB (i.e., gross pathological lesions) observed at post-mortem inspection. The type of bTB lesion was defined as: focal or generalized. Focal lesions were found in one area either in lungs, head, or lymph nodes such as prescapular lymph node, popliteal lymph node, internal iliac lymph node, mesenteric lymph node or superficial inguinal lymph node. Generalized lesions were recorded when there were very extensive bTB visible lesions in one region of the carcass or visible lesions in multiple locations. Visible lesions in the liver were also classified as generalized bTB due to the high risk of a bTB systemic infection.

#### Laboratory culture

Granulomatous lesions found in any cattle during post-mortem inspection at the FMIB Nasinu abattoir were sampled for submission to the Fiji Veterinary Pathology Lab (FVPL). If there were multiple masses, up to three masses were sampled. For bTB reactors with no visible bTB lesions, a minimum of three samples from lymph nodes were collected: for example, a sample of the lymph nodes from the head, lung and liver, or any other enlarged lymph node.

Records for sampled individual bTB reactors and non-reactors in 2018 and sampled bTB reactors from 2019 and 2020 were obtained from the FVPL. The laboratory records did not distinguish between gross lesions from a bTB reactor or a non-reactor, and it was not possible to verify against any other data source in this study. Since the laboratory was carrying out confirmatory tests for *M. bovis*, during 2019 and 2020, only samples from reactor animals with NVL were cultured to properly allocate the use of limited laboratory reagents and consumables, and samples from bTB reactor and non-reactor animals with gross lesions were not cultured. Samples of visible bTB lesions and NVLs were cultured in Löwenstein-Jensen pyruvate agar and Löwenstein-Jensen glycerol agar. Suspensions from culture plate colonies were prepared on glass slides, Ziehl-Neelsen staining was carried out and acid-fast bacilli were confirmed by light microscopy, enabling a presumptively culture positive classification. The FVPL dataset included: bTB culture line number, date of culture, animal identification number, laboratory case number, farm name, abattoir sample type (NVL, lung bTB, head bTB, popliteal bTB, generalized bTB, liver bTB), Löwenstein-Jensen pyruvate agar result, Löwenstein-Jensen glycerol agar result, Ziehl-Neelsen staining result and date read.

#### Animal movement

For 2019–2020, animal movements were identified based on a unique identity animal being present on different farms. The following types of movements were studied: animal movements from non-clear farms to a farm in a different province; animal movements from non-clear farms to a farm in the same province; animal movements from clear farms to a farm in a different province; and animal movements from clear farms to a farm in the same province.

### Data analysis

All data analysis was performed using R (version 4.0.4).

Budget allocation for each year of the study was calculated as the sum of monthly estimates of the BTEC program operations that included the bovine tuberculosis and brucellosis testing performed during the same farm visit, from January to December.

The number of bTB tests conducted was the sum of the number of tests done each year.

BTEC program coverage for 2019 and 2020 was calculated from the number of unique identity animals with a complete bTB test as numerator and the Fiji Agricultural Census 2020 animal count as denominator. Coverage was tabulated by division and by type of farm operation.

Annual and 6-year figures on the proportions of infected unique identity farms and animals were calculated at national, divisional and provincial levels, and by type of farm operation. Unique identity farms were used to identify previously and newly enrolled farms in the BTEC program and to calculate their bTB apparent prevalence.

Further analysis of dairy farms was done including the detection of bTB by size of dairy farm operation, geographical regions, and the number of bTB test visits. The proportion of licensed dairy farms was investigated using Dairy Inspection department records.

The proportion of incomplete on-farm testing outcomes in 2019 and 2020 was calculated according to the number of farms with untested animals across all the farms tested by BTEC. The proportion of incomplete on-farm testing was tabulated by division and by dairy and beef farm operation types.

For farms tested in 2019 and 2020, the proportion of missing animals was calculated based on the median number of animals tested and missing per BTEC testing visit during each year.

From 2015 to 2017, the unique identity animal number from BTEC Meat Compensation records was used to count the number of reactor animals sent to the abattoir; all duplicated unique identity animal numbers were excluded (Figure 1). From 2018 to 2020, the unique identity animal number was used to count the number of reactor animals sent to the abattoir with the one following exception: in the event of a duplicated unique identity animal among the meat certificate records (Figure 1), a detailed check of the carcass weight, type of lesion detected, and meat certificate number was undertaken, and if all identical one of the duplicated records was deleted, but if different the separate records were retained.

The proportion of positive bTB cultures was calculated for reactors with NVL.

During 2019 and 2020, unique identity animals that were present on more than one farm were used to calculate the proportion of animal movements from bTB clear and non-clear farms.

## Results

The available data on bTB included in this study cover the period 2015 to 2020 and so includes periods during and after implementation of the new SOPs from 2014 and the enhancements to *Bovibase* from 2019.

### Budget and bTB testing

The cumulative budget for BTEC program operations addressing bTB and brucellosis from 2015 to 2020 was 5.06 M USD (Table 1) with an average of 0.84 M USD budget per year. There were increases to the budget of 47.4% in 2018 and 32.1% in 2019. The increase in annual funding was associated with an increase in testing. For 2018, the apparent lower number of tests was due to missing bTB testing farm records.

**Table 1:** Budget and number of tests performed by the BTEC program in Fiji.

**Year**	**BTEC budget (USD)**	**Number of bTB tests**
2015	482,279	18,986
2016	482,279	17,252
2017	482,279	28,309
2018	916,310	25,566
2019	1,350,340	37,020
2020	1,350,340	45,244
**Total**	5,063,827	172,377

### National, divisional, and provincial coverage of Fiji's BTEC program 2015–2020

From 2015 to 2020, a total of 3995 farm visits were conducted by the BTEC program across the 4 Divisions of Fiji. This corresponded to 1,280 unique identity farms, the vast majority of which were tested more than once in this period. On average 172 new farms (median 209; range 78–236) were enrolled each year from 2016–2020. On average, 21,560 cattle (median 21,481; range 13,894–28,616) from 455 farms (median 474; range 217–648) were tested per year during the 6 years of this study (Table 2).

**Table 2:** Number of unique identity farms and animals tested each year for bovine tuberculosis by the Fiji BTEC program from 2015 to 2020.

**Year**	**No. farms^a^**	**No. animals^a^**	**No. newly enrolled farms**	**Positive tests**			
				**Farms**		**Animals**	
				**No.^a^**	**%**	**No.^a^**	**%**
2015	331	17176	-	59	17.8	739	4.3
2016	217	13894	78	78	35.9	1130	8.1
2017	483	22609	236	114	23.6	725	3.2
2018	465	20353	209	107	23.0	631	3.1
2019	587	26710	222	200	34.1	1095	4.1
2020	648	28616	205	227	35.0	899	3.1
Total tested	2731	129358		785		5219	
Total unique identity tested	1280	83602		442	34.5	5198	6.2

^a^No. of unique identity farms or animals tested or with positive results in that year; many farms and some animals were tested more than once per year; the same farm or animal can appear in more than one year; the total number of unique identity farms and animals are shown in the last row.

A total of 83,602 individual animals were tested during the 6 years of the study with some of them tested more than once according to the BTEC unique identity animal numbers from 2015 to 2020. This number might underrepresent or overrepresent the total number of individual animals tested before 2018, because during this period a hard copy list of animal ID numbers to be tested was not taken on each farm test visit and no record of lost metal ear tags or replacement ear tag ID numbers was kept. It is also likely that there were missing hard copies of field sheets for 2018.

**Figure 3:**
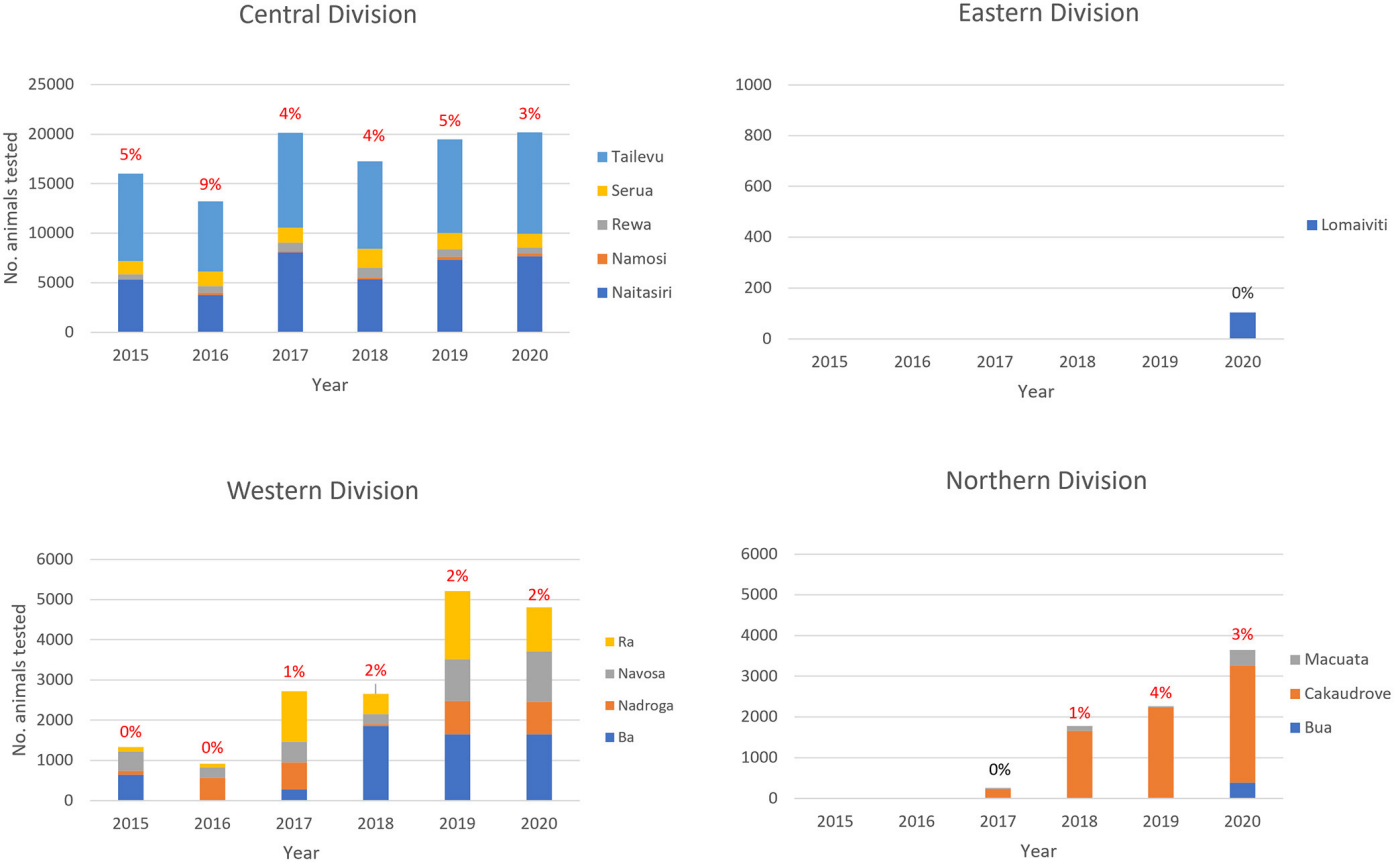
Total number of unique identity cattle tested each year by division and province in the Fiji BTEC program from 2015 to 2020. The percentage positive for bovine tuberculosis is shown above each bar.

Central and Western Division were visited each year from 2015, while testing commenced in Northern division from 2017 and Eastern division in 2020, so that 2020 was the only year in which all 4 divisions were visited (Figure 3). All provinces were consistently tested in Central Division and in Western Division. For Northern Division, all provinces were included only in 2017 and 2020 compared to just one province out of four in Eastern Division.

The majority of animals tested were in Central Division with an average of 17,373 cattle tested per year between 2015 - 2020 (median 17,744; range 13,008–20,099). Then, in decreasing order of total number of animals tested were: Western Division (median 2,654; range 913–5,201) tested in 6 years, Northern Division (median 2,022; range 254–3,646) tested in 4 years, and the Eastern Division with only 104 animals tested in 2020 (Figure 3).

#### Coverage

From 2019 to 2020, based on the 2020 FAC, BTEC had a greater average coverage of dairy cattle (30.3%) than beef cattle (19.1%) across Fiji, and a greater average coverage of cattle in the Central Division (59.8%) compared to Western (8.0%), Northern (13.5%) and Eastern Divisions (2.5%) (Table 3). In Central Division, on average 72.5% of the dairy cattle were tested (Table 3). In 2020, a greater number of beef farms were tested than in previous years due to higher enrolment of new farms from this sector and extension of the BTEC program (Table 4).

**Table 3:** BTEC coverage of beef cattle, dairy cattle, and the total cattle population in Fiji by division in 2019 and 2020. All data were unique identity.

**Year**	**Division**	**No. beef cattle**			**No. dairy cattle**			**Total cattle population** ^**a**^		
		**Census**	**BTEC**	**Coverage (%)**	**Census**	**BTEC**	**Coverage (%)**	**Census**	**BTEC**	**Coverage (%)**
2019	Central	12,924	5,235	40.5	20,093	14,163	70.5	33,017	19,350	58.6
	Western	40,244	4,181	10.4	22,310	650	2.9	62,554	5,201	8.3
	Northern	15,265	2,210	14.5	6,744	-	0.0	22,009	2,269	10.3
	Eastern	1,608	-	0.0	503	-	0.0	2,111	-	0.0
	Total	70,041	11,626^b^	16.6	49,650	14,813	29.8	119,691	26,820^b^	22.4
2020	Central	12,924	5,225	40.4	20,093	14,967	74.5	33,017	20,099	60.9
	Western	40,244	4,337	10.8	22,310	362	1.6	62,554	4,809	7.7
	Northern	15,265	3,611	23.7	6,744	-	0.0	22,009	3,646	16.6
	Eastern	1,608	104	6.5	503	-	0.0	2,111	104	4.9
	**Total**	70,041	13,277^b^	19.0	49,650	15,329	30.9	119,691	28,658^b^	23.9

^a^This total includes beef cattle, dairy cattle and a small number of cattle at holding facilities (n = 381 in 2019; n = 52 in 2020). ^b^This total is the sum of the unique identity animals by division. Animals that moved between divisions in a calendar year and were tested in each division would be counted twice. Thus, these totals are slightly higher than in Tables 2, 4.

**Table 4:** Beef farms, dairy farms and holding facilities: Number of tests, number of farms and animals tested for bTB by the Fiji BTEC program from 2015 to 2020.

**Farm Type**	**Number of unique identity farms**	**Year**	**Number of**	**Number of**		**Number of**	**Positive results**			
	**across the study period ^b^**		**tests**				**Farms**		**Animals**	
				**Farms tested**	**Animal tested**	**New farms**	**No**.	**%**	**No**.	**%**
Beef	822	2015	2870	78	2860	-	9	11.5	31	1.1
		2016	3510	70	3101	55	18	25.7	165	5.3
		2017	9849	230	8013	184	36	15.7	106	1.3
		2018	7927	202	6946	148	41	20.3	133	1.9
		2019	13633	284	11606	176	78	27.5	413	3.6
		2020	17575	355	13270	185	113	31.8	386	2.9
Dairy	435	2015	16105	252	14434	-	50	19.8	708	4.9
		2016	13711	144	10901	21	60	41.7	966	8.9
		2017	18227	248	14844	50	77	31.0	619	4.2
		2018	17158	246	13650	48	63	25.6	491	3.6
		2019	22826	285	14808	43	117	41.1	674	4.6
		2020	27447	276	15327	23	113	40.9	512	3.3
Holding facilities ^a^	23	2015	11	1	11	-	-	-	-	-
		2016	31	3	29	2	-	-	-	-
		2017	233	5	223	2	1	20.0	1	0.4
		2018	477	16	399	12	3	18.8	9	2.3
		2019	561	18	546	4	5	27.8	8	1.5
		2020	222	17	222	2	1	5.9	1	0.5

^a^Holding facility: a site designated for holding cattle for short periods of time such as impounded straying cattle with and without ear tags. ^b^Total number of unique identity farms that were visited by BTEC from 2015 to 2020. In the columns to the right, data are the number of tests applied, the unique identity farms or animals tested, and the farms or animals with positive results in that year. Many farms and some animals were tested more than once per year; the same farm or animal can appear in more than one year, but within a year a farm or animal appears only once.

Before 2015, holding facilities were not visited by BTEC, but during the study period and with the increased restrictions on movement of untested cattle, farmers reclaiming their cattle from holding facilities requested testing of their animals before return to their farm. The number of bTB tests conducted in holding facilities increased gradually from 2015 to 2019, but in 2020 the number of tests decreased because animals in these facilities were sent directly to the abattoir for slaughter (Table 4).

### Detection of bTB nationally and by division and province

A total of 5,219 bTB positive tests were identified nationally from 2015 to 2020, corresponding to 5,198 unique identity animals (21 of which had multiple tests) from a total population of 83,602 (6.2%) cattle that were tested (Table 2). This represents an overall reactor rate or apparent prevalence of 6.2%. The reactors came from 442 unique identity farms, which represented 34.5% of the total of 1280 farms that were tested.

On average, 870 cattle (median 819; range 631–1130) from 131 farms (median 111; range 59–227) tested positive per year during the study. bTB positive cattle were identified in 3 of 4 geographic divisions, and in these 3 divisions testing was performed consistently over 4 or more consecutive years (Figure 3).

#### Central division

Between 2015 and 2020, a total of 4,724 unique identity cattle out of a total population of 64,663 (7.3%) that were tested were detected as bTB reactors in Central Division. They came from 336 unique identity farms, which represented 38.3% of the total of 877 unique identity farms that were tested.

For Central Division, testing was conducted each year in the 5 provinces (Naitasiri, Namosi, Serua, Tailevu, Rewa) (Supplementary Table 2). On average, a total of 791 cattle (median 721; range 576–1127) from 110 farms (median 95; range 56–178) tested positive to bTB each year during the study period.

bTB positive cattle were detected consistently in all provinces, but particularly in Tailevu and Naitasiri, which had the most reactors during the study. In Tailevu, on average 554 cattle (median 495; range 433–791) from 51 farms (median 47; range 33–75) tested positive each year. In Naitasiri, on average 163 cattle (median 151; range 68–322) from 45 farms (median 37; range 19–79) tested positive to bTB each year. In Serua, on average 45 cattle (median 32; range 12–111) from 8 farms (median 7; range 2–16) tested positive to bTB each year. In Rewa, on average 27 cattle (median 29, range 1–58) from 5 farms (median 5, range 1–7) tested positive to bTB each year. In Namosi, on average 4 cattle (median 1, range 1–8) from one farm (median 1, range 1–3) were bTB positive each year.

#### Western division

Between 2015 and 2020, a total of 250 unique identity cattle out of a total population of 14,385 (1.7%) that were tested were detected as bTB reactors in Western Division. They came from 85 unique identity farms, which represented 24.6% of the total of 346 unique identity farms that were tested.

For Western Division, testing was conducted from 2015 to 2020 in the 4 provinces (Ba, Nadroga, Navosa and Ra) (Supplementary Table 3).

#### Northern division

Between 2017 and 2020, a total of 225 unique identity cattle out of a total population of 5,557 (4.1%) that were tested were detected as bTB reactors in Northern Division. They came from 21 unique identity farms, which represented 42.0% of the total of 50 unique identity farms that were tested.

For Northern Division, testing was conducted from 2017 to 2020 across its 3 provinces (Bua, Cakaudrove and Macuata) (Supplementary Table 4).

#### Eastern division

Cattle in the Eastern Division (Lomaiviti province) were tested only in 2020, with no bTB positives detected from among the 140 cattle tested.

### Detection of bTB by type of farm operation

Overall, between 2015 and 2020, bTB was detected on 24% of beef farms, 54% of dairy farms and 35% of holding facilities that were tested, with a significantly higher proportion of bTB positive dairy farms than beef farms (*P* < 0.0001). In beef farms, on average 206 cattle (median 149; range 31–413) from 49 farms (median 39; range 9–113) tested positive to bTB each year (Table 4). In dairy farms, on average 662 cattle (median 647; range 491– 966) from 80 farms (median 70; range 50–117) tested positive to bTB each year. On average 5 cattle (range 1–9) from 3 holding facilities (range 1–5) tested positive to bTB each year from 2017 to 2020 (Table 4).

From 2015 to 2020, the proportion of bTB positive beef farms was similar across Central Division and Western Division, ranging from around 10 to 30% each year (Table 5). For dairy farms, higher proportions of bTB infected farms were found each year in Central Division (range 20 to 44%) compared to Western division (range 13.3–24%). In Central Division, bTB positive cattle were detected in approximately 2 of 4 holding facilities each year from 2017 to 2020. In Western Division, in approximately 1 of 10 holding facilities contained bTB infected cattle from 2018 to 2020 while in Northern Division, approximately 2 of 4 holding facilities contained bTB infected cattle during that period (Table 5).

**Table 5:** Number of farms tested by the BTEC program and proportion of bTB positive by farm type from 2015 to 2020 across the four Divisions of Fiji.

**Division**	**Number of unique identity farms**	**Year**	**Beef farms**		**Dairy farms**		**Holding facilities**	
	**across the study period ^a^**		**Total**	**Positives (%)**	**Total**	**Positives (%)**	**Total**	**Positives (%)**
Central	532 beef farms	2015	63	8 (12.7)	237	48 (20.3)	1	-
	339 dairy farms	2016	58	17 (29.3)	143	60 (42.0)	1	-
	6 holding facilities	2017	182	32 (17.6)	244	77 (31.6)	1	1 (100.0)
		2018	125	25 (20.0)	206	54 (26.2)	2	1 (50.0)
		2019	187	53 (28.3)	239	106 (44.4)	4	2 (50.0)
		2020	236	69 (29.2)	253	109 (43.1)	6	-
Western	238 beef farms	2015	15	1 (6.7)	15	2 (13.3)	-	-
	96 dairy farms	2016	12	1 (8.3)	1	-	2	-
	12 holding facilities	2017	42	4 (9.5)	4	-	4	-
		2018	68	13 (19.1)	40	9 (22.5)	10	1 (10.0)
		2019	89	23 (25.8)	46	11 (23.9)	10	1 (10.0)
		2020	75	27 (36.0)	23	4 (17.4)	8	1 (12.5)
Northern	45 beef farms	2015	-	-	-	-	-	-
	5 holding facilities	2016	-	-	-	-	-	-
		2017	6	-	-	-	-	-
		2018	9	3 (33.3)	-	-	4	1 (25.0)
		2019	8	2 (25.0)	-	-	4	2 (50.0)
		2020	37	17 (45.9)	-	-	3	-
Eastern	7 beef farms	2020	7	-	-	-	-	-

^a^Total number of unique identity farms visited by BTEC from 2015 to 2020. In the columns to the right, data are the farms tested with positive results in that year. Many farms were tested more than once per year; the same farm can appear in more than one year, but within a year a farm appears only once.

### bTB on previously and newly enrolled farms

**Figure 4:**
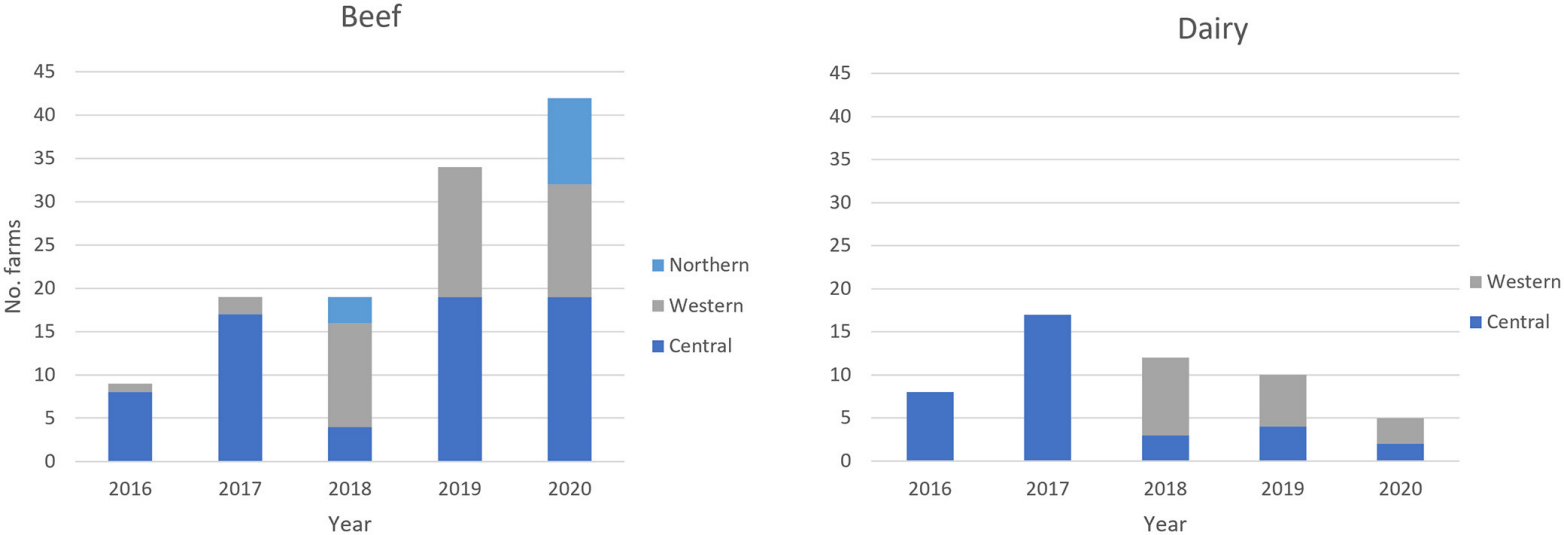
Geographic location of the bTB positive newly enrolled beef and dairy farms from 2016 to 2020.

Previously untested farms were enrolled in the BTEC program each year. The newly enrolled beef farms with bTB positive animals were located across Western Division and Central Division from 2016 to 2020, and in Northern Division in 2018 and 2020. The newly enrolled dairy farms with bTB positive animals were found in Central Division from 2016 to 2020 and from 2018 onwards in Western Division (Figure 4).

### Dairy farms

#### Prevalence

Between 2015 and 2020, a total of 3,956 unique identity cattle out of the total of 52,159 [7.6%, 95% CI (7.36, 7.81)] that were tested were detected as bTB reactors on dairy farms. They came from 234 unique identity dairy farms, which represented 53.8% of the total 435 unique identity farms that were tested.

Dairy farms were covered by the BTEC program only in Central Division and Western Division (Table 5). In Central Division, bTB positive cattle were found each year from 2015 to 2020 and in the Western Division in 2015, 2018, 2019 and 2020.

In Central Division, between 2015 and 2020, a total of 3,912 unique identity dairy cattle out of a total of 50,237 (7.79%) that were tested were detected as bTB reactors on dairy farms. They came from 209 unique identity dairy farms, which represented 61.65% of the total 339 unique identity farms that were tested. On average, a total of 76 farms (median 69; range 48–109) tested positive each year (Table 5).

In Western Division, between 2015 and 2020, a total of 44 unique identity dairy cattle out of a total of 1,985 (2.2%) that were tested were detected as bTB reactors on dairy farms. They came from 25 unique identity dairy farms, which represented 26.0% of the total 96 unique identity farms that were tested. On average, a total of 7 farms (median 7; range 2–11) tested positive each year (Table 5).

#### Detection of bTB by size of dairy farm operation and division

Overall, between 2015 and 2020, according to unique identity farm data, bTB was detected on 87.6% of commercial farms, 58.6% of semi-commercial farms and 40.2% of subsistence farms in Central Division. Overall, of the three sizes of farm operations, the proportion of bTB positive commercial farms was significantly higher (*P* < 0.0001) than the other sizes. For Central Division, from 2015 to 2020, on average, 559 cattle (median 530; range 324–667) from 41 commercial farms (median 40, range 28–58), 63 cattle (median 68; range 20–93) from 25 semi-commercial farms (median 21, range 12–44), and 33 cattle (median 18; range 8–113) from 9 subsistence farms (median 9, range 8–11) were detected bTB positive each year during the study (Table 6).

**Table 6:** Dairy farms in Central division by operation size: Number of tests, number of farms and animals tested for bTB by the Fiji BTEC program from 2015 to 2020.

**Operation size**	**Number of unique identity farms across the study period^a^**	**Year**	**Number of**	**Number of**		**Number of**	**Number and percentage with positive results**			
			**tests**				**Farms**		**Animals**	
				**Farms tested**	**Animal tested**	**New farms**	**No**.	**%**	**No**.	**%**
Commercial	97	2015	11660	76	10236	-	28	36.8	667	6.5
≥ 41 cattle		2016	11351	58	8833	8	36	62.1	893	10.1
		2017	12816	77	10280	9	43	55.8	503	4.9
		2018	11743	71	9538	1	29	40.8	324	3.4
		2019	17147	82	10284	2	58	70.7	556	5.4
		2020	20736	84	10759	1	54	64.3	408	3.8
										
Semi-commercial	145	2015	3075	99	2994	-	12	12.1	20	0.7
≥ 16– ≤ 40 cattle		2016	1886	53	1736	6	15	28.3	56	3.2
		2017	4326	108	3840	19	24	22.2	91	2.4
		2018	3724	91	3065	6	17	18.7	37	1.2
		2019	4338	111	3430	10	40	36.0	93	2.7
		2020	5367	116	3631	5	44	37.9	79	2.2
Subsistence	97	2015	956	62	952	-	8	12.9	18	1.9
≥ 1– ≤ 15 cattle		2016	466	32	460	6	9	28.1	18	3.9
		2017	1026	59	913	18	10	16.9	25	2.7
		2018	925	44	759	6	8	18.2	113	14.9
		2019	687	46	570	1	8	17.4	8	1.4
		2020	977	53	682	4	11	20.8	18	2.6

^a^Total number of unique identity farms that were visited by BTEC from 2015 to 2020. In the columns to the right, data are the number of tests applied and the unique identity farms or animals tested or with positive results in that year. Many farms and some animals were tested more than once per year; the same farm or animal can appear in more than one year, but within a year a farm or animal appears only once.

**Figure 5:**
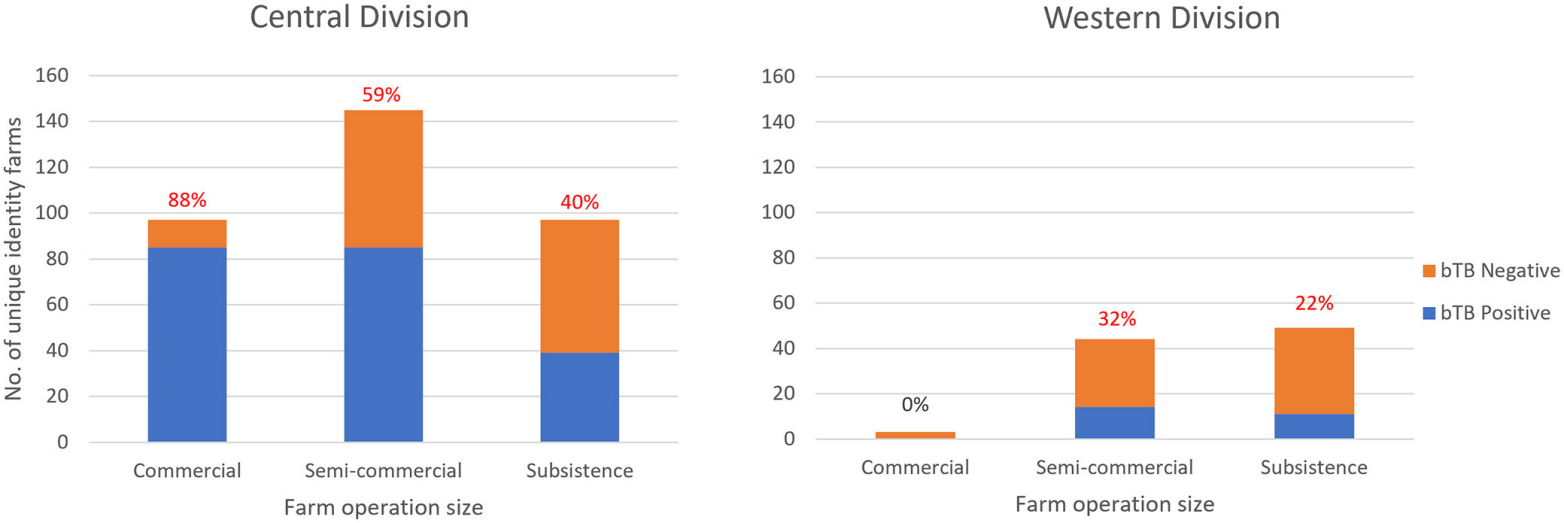
The number of bTB negative and positive unique identity farms classified by operation size, based on tests conducted by the Fiji BTEC program between 2015 and 2020. The % of farms with bTB reactors are shown above each bar.

For Western Division, bTB was detected on 31.8% of semi-commercial farms and 22.4% of subsistence farm; no bTB reactors were detected on commercial farms (Figure 5).

#### Farm bTB status

In 2019 and 2020, only 21.3% (51/239) of the tested dairy farms maintained their clear status, another 8.4% (20/239) reverted to infected after one year or more of being bTB clear, and most farms remained infected during these 2 years. Five of the farms that reverted to infected status had untested cattle during the time that they were considered to be clear from bTB.

#### Untested animals

Untested animals were recorded only during 2019 and 2020. Before this, the farmer may have informed the BTEC team of missing animals but there was no way to capture the information. In 2019, the proportion of farms with untested animals in Central Division was higher in Naitasiri and Tailevu provinces with similar distribution across beef and dairy cattle (Table 7).

**Table 7:** Proportion of beef and dairy farms in Central Division with untested animals in 2019 and 2020.

			**Farms 2019**						**Farms 2020**					
**Division**	**Number of unique identity animals untested^a^**	**Province**	**Total No**.		**Farms with non-tested cattle** ^**b**^				**Total No**.		**Farms with non-tested cattle** ^**b**^			
			**Beef**	**Dairy**	**Beef**	**%**	**Dairy**	**%**	**Beef**	**Dairy**	**Beef**	**%**	**Dairy**	**%**
Central	1561	Naitasiri	61	110	21	34.4	68	61.8	92	117	34	37.0	68	58.1
		Namosi	6	0	1	16.7	-	-	7	0	2	28.6	-	-
		Rewa	17	13	4	23.5	-	23.1	16	13	1	6.3	-	-
		Serua	38	15	3	7.9	9	60.0	35	16	13	37.1	9	56.3
		Tailevu	69	102	17	24.6	23	22.5	91	109	18	19.8	53	48.6

^a^Total number of unique identity animals untested from 2019 to 2020.

^b^Farms with at least one animal without a complete bTB test.

#### Missing animals

Missing animals were recorded only during 2019 and 2020. An increase in the number of reported missing animals occurred in 2020 across all farm sizes of operation.

In 2019 and 2020, on average, the commercial farm category had the highest number of missing cattle, being 1,729 (21.2%) cattle from 67 (80.5%) farms compared to 895 (30.2%) from 79 (68.1%) semi-commercial farms and 167 (24.7%) from 34 (64.6%) subsistence farms (Supplementary Table 5).

### bTB lesion detection at the abattoir

**Figure 6:**
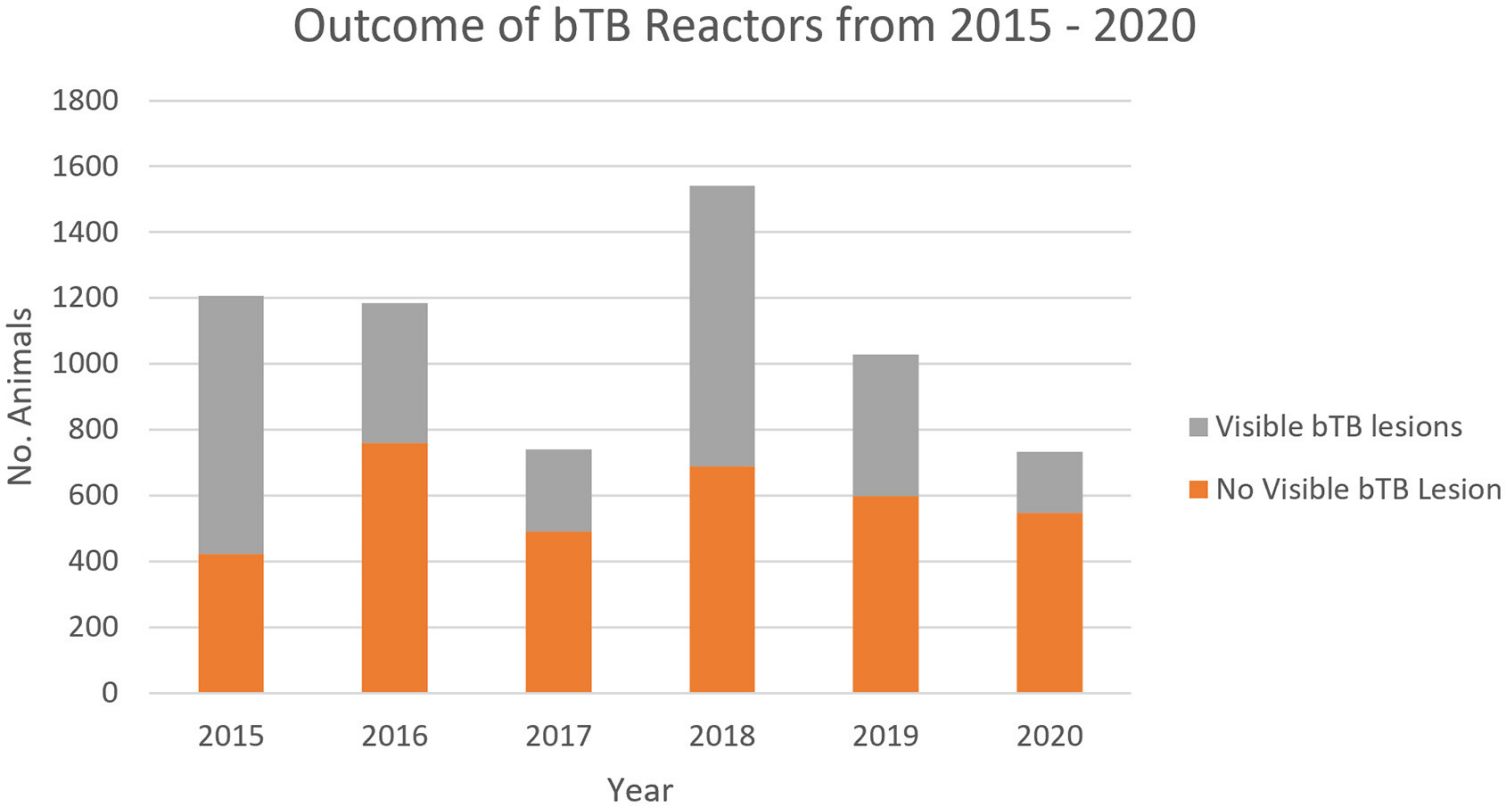
Post-mortem designation of all reactor animals sent to FMIB Nasinu abattoir.

The highest numbers of reactors with visible lesions were found in 2015 and 2018 when there were 784 and 852 animals, respectively, while the lowest numbers of reactors with visible lesions were found in 2017 and 2020 when there were 250 and 186, respectively. The number of animals with NVL fluctuated considerably between years, ranging from 423 to 760. From 2018 to 2020, there was a decrease in the proportion of reactors with visible lesions compared to NVL (Figure 6).

### bTB laboratory diagnostics

In 2018, the proportion of bovine tissue samples (visible lesions and NVL) sent to the laboratory that were presumptively positive for M. bovis by culture was 66.7%. In 2019 and 2020, only samples from NVL reactors were sent to Fiji Veterinary Laboratory for culture. The percentage of samples positive for bacterial isolation in Löwenstein-Jensen pyruvate agar was 90% (463/512) in 2019, and 68% (354/520) in 2020 (Supplementary Table 6). During those years, on average 79% were presumptively culture positive for *M. bovis*. In 2018, 66.2% of the cultures from the NVL reactors were positive, i.e., a lower percentage compared to 2019 and 2020 results. In 2019, 14 bacterial cultures in Löwenstein-Jensen pyruvate were submitted for *Mycobacterium* species identification by PCR and 92.8% were confirmed to be positive for *M. bovis*.

### Animal movements

#### Movements from non-clear farms

##### To a farm in a different province

In 2019, most of the animals that were moved from non-clear farms to any other farm between different provinces were sent to Central Division. Ninety-five farms in 9 provinces and 3 divisions sent a total of 153 animals to 83 farms in Central Division. Eventually, three of these moved animals were identified to be bTB positive and slaughtered at FMIB Nasinu abattoir in Central Division (Table 8).

**Table 8:** Animal movements from non-clear farms to a farm in a different province and from clear farms to a farm in a different province during 2019–2020.

	**Movement origin**	**Movement destination**				
**Year**	**Division: Province**	**Division: Province**	**Animals tested** ^**a**^		**Farms tested** ^**a**^	
			**Total population**	**No. moved (%)**	**Total population**	**No. with movements (%)**
**Animal movements from non-clear farms**						
2019	**Central:** Naitasiri, Namosi, Serua, Rewa, Tailevu. **Western:** Ba, Nadroga, Ra. **Northern:** Cakaudrove	**Central:** Naitasiri, Namosi, Serua, Rewa, Tailevu	19,350	153 (0.8)	430	83 (19.3)
						
	**Central:** Naitasiri, Serua, Tailevu. **Western:** Ba, Nadroga. **Northern:** Cakaudrove	**Western:** Ba, Nadroga, Navosa, Ra	5,201	44 (0.8)	145	24 (16.6)
						
	**Central:** Naitasiri, Tailevu. **Western:** Ra	**Northern:** Cakaudrove	2,269	6 (0.3)	12	2 (16.7)
						
2020	**Central:** Naitasiri, Serua, Tailevu. **Western:** Ba	**Central:** Naitasiri, Serua, Tailevu	20,099	34 (0.2)	495	13 (2.6)
						
	**Central:** Tailevu	**Western:** Ba	4,809	2 (0.04)	106	2 (1.9)
**Animal movements from clear farms**						
2019	**Central:** Naitasiri, Rewa, Serua Tailevu **Western:** Navosa	**Central:** Naitasiri, Namosi, Serua, Rewa, Tailevu	19,350	17 (0.1)	430	14 (19.3)
						
	**Central:** Namosi, Tailevu **Western:** Navosa	**Western:** Ba, Navosa, Ra	5,201	5 (0.1)	145	4 (2.8)
						
	**Central:** Tailevu	**Northern:** Cakaudrove	2,269	2 (0.1)	12	2 (16.7)
2020	**Central:** Naitasiri, Tailevu	**Central:** Naitasiri, Rewa, Tailevu	20,099	9 (0.04)	495	4 (0.8)

^a^Number of unique identity animals and farms involved in movements.

In 2020, there was an apparent reduction in such movements with only thirteen farms in 4 provinces and 2 divisions moving a total of 34 animals to 13 farms in Central Division (Table 8). Eventually, one of the moved animals tested positive and was sent to slaughter at FMIB Nasinu abattoir in Central Division.

In 2019, of the animals tested in each division, 0.8% of animals in Central Division, 0.8% in Western Division and 0.3% in Northern Division had been moved from non-clear farms in different provinces in the same or a different division. However, in 2020, such movements decreased to 0.2% from Central Division and 0.04% from Western Division (Table 8).

##### To a farm in the same province

In 2019, most of the animals that were moved from non-clear farms to any other farm in the same province occurred in Central Division. In total, 39 localities moved 263 animals across 102 farms in the Central division. Eventually, fifteen of these moved animals—two that tested positive to bTB before being moved and thirteen that subsequently tested positive to bTB—were sent slaughter at FMIB Nasinu abattoir in Central Division (Table 9). Of the animal population tested in each province, 2.1% from Naitasiri, 0.3% from Rewa, 0.2% from Serua and 1.1% from Tailevu province came from non-clear farms (Table 9).

**Table 9:** Animal movements from non-clear farms to a farm in the same province and from clear farms to a farm in the same province during 2019-2020.

**Year**	**Division**	**Province**	**Animals tested** ^**a**^		**Farms tested** ^**a**^	
			**Total no**.	**No. moved (%)**	**Total no**.	**No. moved (%)**
**Animal movements from non-clear farms**						
2019	Central 263 animals, 102 farms, 39 localities	Naitasiri	7332	153 (2.1)	169	54 (32.0)
		Rewa	755	2 (0.3)	31	2 (6.5)
		Serua	1675	4 (0.2)	53	5 (9.4)
		Tailevu	9413	104 (1.1)	171	41 (24.0)
	Western 7 animals, 6 farms, 6 localities	Ba	1646	3 (0.2)	110	3 (2.7)
		Nadroga	838	1 (0.1)	10	1 (10.0)
		Ra	1693	3 (0.2)	11	2 (18.2)
	Northern 4 animals, 3 farms, 2 localities	Bua	23	1 (4.3)	4	1 (25.0)
		Cakaudrove	2215	3 (0.1)	5	2 (40.0)
2020	Central 164 animals, 47 farms, 21 localities	Naitasiri	7668	119 (1.6)	206	22 (10.7)
		Tailevu	10243	45 (0.4)	202	25 (12.4)
**Animal movements from clear farms**						
2019	Central 59 animals, 33 farms, 18 localities	Naitasiri	7332	16 (0.2)	169	11 (6.5)
		Tailevu	9413	43 (0.5)	171	22 (12.9)
2020	Central 128 animals, 11 farms, 7 localities	Naitasiri	7668	6 (0.1)	206	6 (2.9)
		Tailevu	10243	122 (1.2)	202	5 (2.5)

^a^Number of unique identity animals and farms involved in movements.

In Western Division, in total, 6 localities across Ba, Nadroga and Ra provinces moved 7 animals to 6 farms. In Northern Division, in total, 2 localities across Bua and Cakaudrove provinces moved 4 animals to 3 farms (Table 9).

In 2020, animal movements from non-clear farms within a province were registered only in Central Division. In total, 21 localities across Naitasiri and Tailevu provinces, moved 164 animals to 47 farms in Central Division. Of the animal population tested, 1.6% from Naitasiri and 0.4% from Tailevu were moved between non-clear farms. Eventually, four of these moved animals- two that tested positive to bTB before being moved, and two that subsequently tested positive to bTB—were sent slaughter at FMIB Nasinu abattoir in Central Division (Table 9).

#### Movements from clear farms

##### To a farm in a different province

In 2019, most of the animals moved from clear farms to any other farm between different provinces were sent to Central Division. Eleven farms in 5 provinces and 2 divisions sent a total of 17 animals to 14 farms in Central Division. None of these animals subsequently tested positive to bTB or were sent to slaughter at FMIB Nasinu abattoir (Table 8). In 2020, there was an apparent reduction in such movements with only 3 farms in 2 provinces and 1 division moving 9 animals to 4 farms in Central Division (Table 8). None of these animals subsequently tested positive to bTB or were sent to slaughter at FMIB Nasinu abattoir.

##### To a farm in the same province

In 2019, all the animal movements from clear farms to any other farm in the same province occurred in Central Division. In total, 18 localities moved 59 animals across 33 farms in Central Division. Eventually, 1 animal moved across Naitasiri province, subsequently tested positive to bTB, and was sent for slaughter at FMIB Nasinu abattoir in Central Division (Table 9). In 2020, 7 localities across Naitasiri and Tailevu provinces moved 128 animals to 11 farms. Eventually, two animals that were moved across Tailevu province subsequently tested positive to bTB and were sent for slaughter at FMIB Nasinu abattoir in Central Division (Table 9).

## Discussion

According to Mussman (22), in less-economically developed countries that generally lack a mobile field service with trained veterinarians and auxiliaries, and that also often lack adequate diagnostic facilities, a disease control programme should have two stages. The first is short-term and includes the development of diagnostic and field services, and training of personnel to deal with diseases and implement control actions. The second is long-term and includes establishment of disease reporting systems and facilities for field surveys and use of the data from these for economic and epidemiological modeling to determine the benefits arising from the control program. To underwrite long-term implementation of an animal disease control program, there must be supporting legislation, political will to enforce the legislation and ongoing, dedicated allocation of government funds for the program. Given the lengthy incubation period for bTB, a control program must be sustained with strict adherence to protocols for an extended period and data be analyzed regularly in order to track progress for return on investment and modification of diagnostic protocols as prevalence reduces. This level of sustained commitment is a serious challenge, particularly in a resource limited context (23–26). But it can lead to absence of bTB disease, as seen in the United States with implementation of control from 1917 achieving prevalence reduction from 5% to < 0.006% in 2011 (27) and even pathogen eradication, as achieved by the Australian control program over 27 years from 1970 to 1997 (6, 28).

Factors contributing to persistent bTB infections are common to all countries and revolve around there being persistent sources of *M. bovis*, but the details will vary from country to country. In Fiji, untested cattle and uncontrolled cattle movements were important contributors, whereas a wildlife reservoir has not yet been identified (29). Studies from Great Britain, the Republic of Ireland, Spain and Uruguay have associated these factors with bTB breakdowns (30–32). To significantly reduce bTB prevalence over time, South American countries such as Argentina, Brazil, Chile and Uruguay intensified their control measures for bTB based on surveillance at abattoirs, test-cull policy, bTB-free farm certification and disease notification (33). Most importantly, in these countries, a national livestock traceability system was implemented alongside these measures to control animal movements and to enable traceback from an abattoir to the original infected herd (33, 34). In countries such as England, the Republic of Ireland, New Zealand and USA, control measures for *M. bovis* reservoirs in wildlife have also been important to bring bTB under control (11, 35–37).

The long commitment of time required for the control bTB can be hindered because of inadequate financial support. In South Africa, for example, re-prioritization of diseases and budget constraints led to a reduction in cattle testing which eventually produced re-emergence of bTB on commercial farms (24). Furthermore, an economic analysis of the cost of bTB-free certification of farms in Brazil reported that is feasible for larger-scale dairy farms, but for smaller scale, less efficient farmers, their inclusion in BTEC required targeted policies that compensated farmers for the additional costs (25). Thus, the lack of financial support compromises the coverage of the program. In contrast to countries like Fiji where participation in BTEC is mandatory, in some South American countries such as Ecuador, Peru, Bolivia, and Paraguay where bTB prevalence is high, regional campaigns are promoted based on the decisions of farmers to engage, but the bTB test is not mandatory for all cattle in the population. Therefore, the true burden of bTB disease remains unknown due to the lack of routine surveillance data, and it is therefore likely that *M. bovis* will persist in the population (38–40).

With respect to the objective of minimizing reservoirs of *M. bovis*, farmers in Fiji are fortunate that the government has supported BTEC testing, provides compensation for cattle that are culled and mandates testing of the national herd. These high-level objectives will require ongoing investment. In this study an objective analysis of data collected over 6 years was used to identify limitations and strengths in the bTB control program, and this led to important recommendations which are discussed below.

### Limitations of the bTB control program in Fiji

Effective control of bTB requires identification of all infected animals. In Fiji, the coverage of the testing program was relatively low, with only 30% of dairy cattle and 20% of beef cattle being tested in 2019 and 2020. Coverage varied considerably depending on geographic region, was budget-dependent and had increased by 2018 as funding increased. The evidence suggested that bTB is highly prevalent across dairy farms and there was no visible trend downwards. Only one in five of the test negative dairy farms maintained its clear status over time, while most farms remained infected. Abattoir and laboratory data suggested that most bTB reactors in Fiji were truly infected with *M. bovis*.

This study provides insight on the factors contributing to persistent bTB infection on farms in Fiji: the bTB infection history of the farm, the size of the farm operation, the number of untested animals and missing animals and uncontrolled animal movements. Most of these factors were described in the previous BTEC retrospective study in Fiji which led to recommendations that informed the new BTEC strategy that was endorsed in 2018. Given the chronic nature of bTB and the long-term action needed to achieve control, this was expected, but with the recent improvements in data quality, we are now more confident in the designation of these factors.

Farms with only a relatively short period of clear status may still have residually-infected cattle that will contribute to farm bTB recurrence and to local/regional persistence of bTB. It is known that animals present on a farm during a bTB outbreak are likely later in their lives to become highly infectious (41, 42). In this study, 55% (11/20) of the dairy farms that changed from clear to infected status had been infected in earlier years of the study period. The future bTB risk of recurrence increases because of cattle-to-cattle transmission from residually-infected cattle in the herd or on neighboring farms, or due to transmission from the environment, wildlife, or humans, but the relevance of these factors needs to be determined for each farm (43, 44).

Consistent with previous findings in several countries (45, 46), in Fiji the risk of bTB was higher for larger farms and was consistently highest among commercial farms. Among these, some re-commenced bTB testing between 2017–2018 and retained reactors on farm until the start of the new compensation scheme in late 2018; in this context compensation had an unintended consequence. Compliance was higher during 2019 and 2020, with about 90% of reactor animals being sent to slaughter soon after detection.

In extensive farming situations in countries such as Fiji, if a whole herd test is not successfully completed, the untested animals can include some that remain a source of *M. bovis* infection for other animals on the farm. This is a problem in Fiji where many animals were identified as untested either because of an incomplete bTB test or due to not being presented by the farmer for testing. Many more cattle were identified as being missing; these were present in the dataset at one or more testing events but not later, without record of their death, slaughter, or sale. The possible reasons for missing animals include death, slaughter for consumption, sale, change to a new animal ID due to tag loss, incomplete muster or straying off the farm. Only some of the missing animals were likely to have permanently left the farm or been given a new ID; the rest probably remained on the farm and were untested.

Animal movements are known to be important for the spread of *M. bovis* in countries such as Great Britain, the Republic of Ireland and Uruguay (31, 47, 48) and were identified in this study in Fiji where they occurred across divisions and provinces from farms that did not have a bTB clear status. A proportion of these animals subsequently tested positive to bTB. Such movements need to be investigated. If movement restrictions are not imposed, export of infection to other farms will occur. A study by Clegg et al. (41) found that farms that experienced a bTB episode (2 or more reactors to skin test) and introduced animals early during the bTB episode were at significantly greater future bTB risk than farms where animals were introduced later (49).

### Strengths of the bTB control program in Fiji

In Fiji, important improvements occurred in the bTB control program during 2014 to 2020, including for example, implementation of a new protocol for SID test reading and regular training of BTEC field staff, upskilling of meat inspectors, formulation of the new BTEC strategy for diagnostic and field services and endorsement of it by industry stakeholders (19). For the longer-term, Fiji has implemented *Bovibase*, the national recording system for the BTEC program and cattle production, which can provide the data for epidemiological and economic evaluations of progress. Strategically, the BTEC program was centralized to the national office in Suva from 2011 to 2019 to concentrate implementation on the dairy industry. Having achieved an increase in dairy farm participation, the BTEC program is now decentralized with the designated responsibility for conduct of the program returned to the MOA division offices in order to increase coverage, particularly of the beef industry. But with this, it is absolutely essential to maintain the level and proficiency of testing in Central Division in order to build on the gains achieved by the dairy industry and support individual farms to achieve and maintain clear status (50).

Whilst acknowledging these improvements, this study revealed several important aspects of Fiji bTB control that are critical to be acted upon if bTB prevalence is to be reduced; they require long-term commitment by the MOA, BAF and industry to achieve success. These are farmer engagement and compliance; implementation of animal movement regulations; consistent and harmonized use of *Bovibase* in order to implement, monitor and evaluate the control program; and continued collaborative research to inform refinement of diagnostic and control protocols. A third BTEC Stakeholder Forum is warranted now to engage all stakeholders in shared, evidence-based decision making on these issues. In addition to opening cattle trade opportunities between Fiji in the Pacific region, the eventual success of the BTEC program in Fiji will encourage neighboring Pacific countries to initiate bTB surveillance in their respective countries, learning from the strengths and limitations of the Fiji program.

### Recommendations for the bTB control program in Fiji

#### Farmer engagement and compliance

Farmer engagement and compliance in an animal disease control program requires a combination of mechanisms to exist and to be applied consistently with support from government, industry and the community. These mechanisms relate to different factors that influence animal health behavior adoption such as external motivation provided by legislation with a formal requirement for participation and known consequences for non-compliance, and intrinsic motivation arising from sufficient knowledge to understand the risk posed to animal health and to their business, and confidence in ability to participate in and realize a benefit from the control program. Current legislation states the roles of specific government agencies in general animal disease control. It would be strengthened by inclusion of specific clauses specifying the roles of different government agencies and industry stakeholders in the continuous implementation of BTEC program as a One Health collaboration, with well-defined consequences for sector non-compliance.

In balance with legal requirements, government agencies need to provide extension services that educate and support farmers to understand bTB risk, to participate in testing and to adhere to cattle movement restrictions (discussed in next section). The important role of the MOA BTEC and BAF officers must be emphasized; in relation to provision of accurate information on bTB transmission and how regular whole herd testing and reactor removal along with cattle movement restrictions reduces transmission. Alongside the bTB control, the government extension officers need to provide services sought by farmers such as production advice and access to bTB-free replacement stock, and practical assistance to ensure testing of all cattle can occur and reactors be promptly sent to slaughter. For example, lack of on-farm animal handling facilities contributes to incomplete testing and farmers can be encouraged to setup simple holding yards to hold animals for further testing and hold reactors until sent to slaughter. This will assist in lowering the numbers of untested and missing animals. Additionally for compliant farmers with clear farms, it would be beneficial to provide a partial subsidy for fencing to minimize the risk of bTB recurrence posed by straying cattle (30).

To foster trust, respect, and farmer belief in the accuracy of information provided on bTB, MOA and BAF officers need to adhere to the SOPs and at the same time seek to develop a framework for communication with the farming community. It is important to examine the psychosocial factors that might influence the decision of farmers about compliance with the BTEC program. A study in Spain conducted interviews to identify the major themes related to how veterinarians and farmers were affected by their BTEC program. Participants mentioned that some of the weak points of the program were communication flow issues. This combined with the complexity of the bTB epidemiology and gaps in stakeholder knowledge contributed to disbelief and distrust in the bTB control program (51). A study by Robinson on qualitative narratives of bTB in Northern Ireland, concluded that farmers may resist rather than actively cooperate with the state because bTB is seen as just another of many farming life aspects demanding attention. Specters of global market forces, regulations, inspections, paperwork, bad weather, stress and disease overshadow and shape their attitudes and actions (52). In addition to enhancing the agenda at the BTEC forum, approaches to the communication issue in Fiji, could be the implementation of participatory studies, social capital studies, where the involvement of communities and development of trust is essential in defining and prioritizing the bTB problem, and in the development of solutions to service delivery, disease control options and surveillance (53, 54).

#### Regulation of cattle movement

The restriction of animal movement from non-clear farms needs to be ensured across Fiji because many such movements were discovered in this study. Continuous cooperation is needed from BAF to decrease animal movements from infected farms and clear farms that still have a high risk for bTB. Consistent and prompt implementation of penalties by BAF for non-compliance is necessary for bTB control in Fiji. Continuous enhancement of *Bovibase* to encourage the usage of its other components by the MOA Regulatory section, BAF, and FDL will allow for more effective monitoring of cattle movement, BTEC testing and herd production improvements in Fiji.

#### Data systems to monitor and evaluation

There was great complexity in the 2015 to 2020 BTEC data that were used in this study. To prepare the dataset for analysis required enormous effort and time due largely to the inconsistent implementation of a unique farm identification number across BTEC field testing data, abattoir and laboratory data. This delayed BTEC staff in uploading the source data to the new cloud-based platform *Bovibase*. However, the implementation of *Bovibase* has enabled the recording of data on BTEC activities with better identification and follow-up of participating farms and animals, recording of untested and missing animals, and stringent farm bTB status classification. The consistent use of *Bovibase* will play a critical role in harmonizing data between all cattle industry stakeholders and promote confidence in the information received about farms, geographic areas, and cattle movements (43, 55). Continued financial investment, development, and enhancement of *Bovibase* is required by the MOA as the lead agency for development of this national database. Stakeholders, particularly the meat and dairy inspector of the MOA Regulatory Section, BAF and Fiji Dairy Cooperative Ltd., must contribute to the maintenance of Bovibase through the provision of data, entering of data and financial subscriptions as pledged during its planning and development stages.

In terms of data quality, a critical need is the consistent use of a unique farm identifier based on geographical coordinates that is the sole identifier used by all agencies including the MOA Animal Health and Production Department, FMIB abattoirs, Fiji Veterinary Laboratory, the Biosecurity Authority of Fiji, FCDCL and Fiji Dairy Limited. This will enable seamless data sharing and provide timely and analysis-ready data, which is a prerequisite to provide evidence for the evaluation of BTEC policy outcomes for all stakeholders. On farm, individual animal identification also warrants improvement, and the use of RFID devices would provide an electronic id [e-id] linked with *Bovibase*, particularly for the dairy industry, to strengthen the determination of bTB farm status and achieve reliable animal traceability.

Other necessary enhancements are the timely encoding of BTEC field sheets into *Bovibase*, the incorporation of abattoir data for the update of animal status on slaughter to improve the accuracy of missing animal numbers and the bTB status of animals identified at abattoir slaughter inspection to contribute to farm status determination. In addition, an agreed protocol for data management and analysis is essential to enable consistent reporting. Reports should be automatically produced on a set schedule, such as weekly reports for farm field schedules, monthly reports of test results and updated clear farm status list, and quarterly reports for operational and budget purposes. Ideally the MOA field epidemiologists or committed senior officers would liaise with the *Bovibase* developer to refine the processes for data entry and data analysis.

Developing a dedicated research program funded by the government and the cattle industry in collaboration with international agencies will have the benefit of capacity building while providing important evidence to inform decision making on diagnostic and control protocols, and evaluation of the cost-benefit of implementation of bTB control at industry and farm levels. Immediate activities to enhance diagnostic capability and to address the presence of residual infected cattle, could include collaborations in repeating testing over time using SID, enhancing microbiological culture methods, introducing procedures for cell-mediated immune assays (interferon gamma release assay) to be evaluated alongside the SID test and establishing proficiency for PCR testing in-country at the Fiji Veterinary Laboratory. Moving beyond presumptive *M. bovis* culture positive status to confirmed status, discriminating *Mycobacterium* species recovered in culture, ruling out *M. tuberculosis* and strain typing for epidemiological tracing are important objectives. Given the importance of the SID for identification of bTB, data are needed from well-designed research trials to estimate the sensitivity and specificity of the SID test on farms with different bTB prevalence levels in Fiji, which would utilize gross necropsy findings from the abattoir and microbiology/PCR test results from the laboratory as independent tests for bTB (56). Maintenance of accurate records in *Bovibase* over time will enable advanced analyses such as spatio-temporal models of bovine tuberculosis in the cattle population and social network analysis (SNA) to characterize patterns of cattle movement and quantify the role of high-risk farms. Research objectives should be a topic for a 3rd BTEC Forum discussion with all stakeholders.

## Conclusion

Relative to the stages of control reached in the decades-long bTB control programs in high-income countries, Fiji is in the early part of its control efforts. The program has been progressively improved and protocols have been enhanced based on technical analysis and stakeholder review. Reflecting this early phase, and consistent with the available financial and human resources, testing for bTB did not reach the majority of cattle in Fiji during the study period. Testing revealed that bTB was highly prevalent, with about 8% of dairy cattle and 62% of dairy farms infected. Against a high baseline prevalence of bTB, there was no visible downward trend in bTB apparent prevalence over 6 years. The factors contributing to this situation were objectively determined to be persistent infections, which were partly due to the significant number of untested animals and uncontrolled animal movements, and the particular importance of large farms. Data management was critical in the control program and is still evolving. There was complexity in the datasets that were compiled in this study, the lack of a consistent unique farm identification number and individual animal identification problems being responsible for time lost in correlating field-testing data, abattoir and laboratory data. However, the implementation, enhancement, and consistent use of *Bovibase* will play a critical role in harmonizing data. bTB remains a serious concern for the Fiji dairy industry and a public health risk for those that consume unpasteurized milk; there is value in One Health research on zoonotic bTB in high-risk communities, to support the human TB control program in Fiji. Implementation of a control strategy targeting the main contributors to bTB persistence that were identified in this study, along with maintenance of a comprehensive reporting and traceability system, industry awareness, and government support, will enable more farms to achieve and maintain a bTB clear status. Capacity building through scientific research that provides data to underpin policy and procedures in BTEC will be required. As in all countries, control of bTB in Fiji is a long-term objective that must have multiple stakeholder engagement and regular review to measure success.

## Data availability statement

The data analyzed in this study is subject to the following licenses/restrictions. The original contributions presented in the study are included in the article/Supplementary material, further inquiries can be directed to the corresponding author. Requests to access these datasets should be directed to jenny-ann.toribio@sydney.edu.au.

## Author contributions

The proposal for this collaborative research was developed by J-AT, AG, EB, and RW. EB, SS, and VS facilitated collation, encoding and validation of field data and provided local documents for referencing. AG constructed and cleaned the dataset for analysis then performed all analysis and interpretation of results. EB and AR provided information about Bovibase contributing to validation of cleaned data and interpretation of the results. J-AT and RW supervised the analysis, presentation of the results and documentation of the outcomes and findings of the study. All authors contributed to the content and the preparation of this manuscript.

## Funding

The Sydney School of Veterinary Science at the University of Sydney funded costs associated with data collation and validation for this study. J-AT was supported by scholarships from the Secretaría Nacional de Ciencia Tecnología e Innovación (SENACYT) and the Instituto para la Formación y Aprovechamiento de los Recursos Humanos (IFARHU) of the Republic of Panama, and the James Ramage Wright Supplementary Scholarship of the University of Sydney.

## Conflict of interest

The authors declare that the research was conducted in the absence of any commercial or financial relationships that could be construed as a potential conflict of interest.

## Publisher's note

All claims expressed in this article are solely those of the authors and do not necessarily represent those of their affiliated organizations, or those of the publisher, the editors and the reviewers. Any product that may be evaluated in this article, or claim that may be made by its manufacturer, is not guaranteed or endorsed by the publisher.
