# Supplementary material for: Bovine tuberculosis control in Fiji: Retrospective study findings for 2015 to 2020
Source: Front Vet Sci. 2022 Sep 30;9:972120. doi: 10.3389/fvets.2022.972120 (PMC9561621; doi:10.3389/fvets.2022.972120)
Supplement: Supplementary file 1 [file Table_1.docx]

Supplementary Material

**Supplementary Table 1:** Summary of data compiled at national, farm and animal levels.

| **Factor** | **Description** | **Data Source** |
| --- | --- | --- |
| *National data* |  |  |
| Cattle population | Data from geographical location (divisions) by cattle production type (dairy and beef) | 2020 Fiji Agriculture Census |
| *Farm level* |  |  |
| Farm name | Local name | BTEC fieldsheets |
| Unique identity farm | *Bovibase* ID, MOA registration number or village/settlement/town supply description | BTEC fieldsheets |
| Farm location | Division, province, village/settlement, locality | BTEC fieldsheets |
| Farm enrolment | Previously enrolled or newly enrolled by year | BTEC fieldsheets |
| Farm type | Beef, Dairy or holding facility (pounds) | BTEC fieldsheets |
| Farm milk supply number | Registration number of a farm that supplies milk | BTEC fieldsheets |
| Licensed farm | Dairy farm that has registered and paid a license fee to officially sell milk to Rewa Dairy | MOA license records |
| Farm operation size | Commercial, semi-commercial or subsistence | Study calculation |
| Farm status | Infected (4), restricted (3), provisionally clear (2) or clear (1) | BTEC fieldsheets |
| Date of test – month year | Month and Year on which a farm was bTB tested | BTEC fieldsheets |
| Total stock | Total animals on farm on the day of testing and/or reading | Study calculation |
| Stock breakdown | Total number of milking cow, bull, bull calf, cow, dry cow, heifer, heifer calf, steer and unknown. One column for each category | Study calculation |
| Total bTB tested | Total number of animals bTB tested | Study calculation |
| Total bTB reactors | Total number of animals positive to bTB test | Study calculation |
| Total NPD4 | Totals number of animals not presented on the reading date (Day 4). This data is available only for 2019 and 2020. | Study calculation |
| Total Not Tested | Totals number of animals not tested that were presented on Day 4. This data available only for 2019 and 2020) | Study calculation |
| Total missing | Total number of missing animals. An animal not presented on Day 1 and Day 4 during a BTEC visit, without record of death, slaughter, or sale. | BTEC fieldsheets |
| Batch number | Number from on-farm bTB testing field sheet. (Data available only for 2019 and 2020) | BTEC fieldsheets |
| BTEC on farm staff | Injector/Reader, blood collector, ear tagging, encoder. (Data available only for 2019 and 2020) | BTEC fieldsheets |
| Total reactors sent to abattoir | Total number of reactors sent to abattoir | Study calculation |
| *Animal level* |  |  |
| Unique identity animal | Unique ear metal tag animal identification listed on BTEC records. | BTEC fieldsheets |
| Animal type | Milking cow, bull, bull calf, cow, dry cow, heifer, heifer calf, steer or unknown | BTEC fieldsheets |
| Sex | Female or male | BTEC fieldsheets |
| bTB test result | Positive, negative, NPD4 or Not tested | BTEC fieldsheets |
| Reactor removed | Reactor removed and sent to slaughter, yes or no | BTEC and FMIB |
| Date of removal | Date removed | BTEC and FMIB |
| Stock movement number (SMA#) | Record of the movement of all animals. E.g., moving animals from farm to farm or from farm to abattoir | BTEC stock movement advice records |
| Date of slaughter | Date a reactor animal was slaughtered | BTEC Meat Compensation Records, FMIB Reactor Meatworks form and Meat Certificates |
| Type of lesion detected | Non-Visible Lesion (NVL) or Visible bTB lesions (Generalized/GTB/Grapes/Multiple sites bTB-condemned, head bTB, liver bTB – condemned, head and lung bTB, lung bTB, prescapular lymph node, popliteal lymph node and other- specify (e.g.: inguinal, mesenteric, or internal iliac) | BTEC Meat Compensation Records, FMIB Reactor Meatworks form and Meat Certificates |
| Condemnation weight (Kg) | Total weight of carcass condemned (not suitable for human consumption) | BTEC Meat Compensation Records, FMIB Reactor Meatworks form and Meat Certificates |
| Pass weight (Kg) | Total weight of carcass suitable for human consumption. | BTEC Meat Compensation Records, FMIB Reactor Meatworks form and Meat Certificates |
| Meat Certificate number | Final documentation of a slaughtered and compensated animal. | BTEC Meat Compensation Records, FMIB Reactor Meatworks form and Meat Certificates |
| Animal type case number | TB specimen unique lab number | FVPL Specimen Submission forms |
| bTB culture result - Löwenstein-Jensen Pyruvate agar | Positive, Negative | FVPL culture result records |
| bTB culture result - Löwenstein-Jensen glycerol agar | Positive, Negative | FVPL culture result records |
| Ziehl- Neelsen staining results | Positive, Negative | FVPL culture result records |
| Date culture started and date read | Day one of the culture and day of culture reading | FVPL culture result records |
| Animal movement | Unique animal identity being present on different farms | BTEC fieldsheets |

**Supplementary Table 2:** bTB test results in Central Division provinces between 2015 and 2020.

| **Central Division**  **Province^1^** | **Year** |  | **Animals tested^2^** | |  | **Farms tested^1^** | |  |
| --- | --- | --- | --- | --- | --- | --- | --- | --- |
|  |  |  |  |  |  |  |  |  |
|  |  |  | **Total No.** | **No. positive (%)** |  | **Total No.** | **No. positive (%)** |  |
| Naitasiri | 2015 |  | 5336 | 68 (1.3) |  | 146 | 19 (13.0) |  |
| (189 Beef farms, | 2016 |  | 3779 | 160 (4.2) |  | 83 | 32 (38.6) |  |
| 159 Dairy farms and | 2017 |  | 8063 | 141 (1.7) |  | 189 | 42 (22.2) |  |
| 2 Holding Facilities) | 2018 |  | 5359 | 92 (1.7) |  | 108 | 28 (25.9) |  |
|  | 2019 |  | 7332 | 322 (4.4) |  | 169 | 70 (41.4) |  |
|  | 2020 |  | 7668 | 194 (2.5) |  | 206 | 79 (38.3) |  |
|  |  |  |  |  |  |  |  |  |
| Namosi | 2015 |  | - | - |  | - | - |  |
|  | 2016 |  | 201 | 8 (4.0) |  | 1 | 1(100) |  |
| (10 Beef farms) | 2017 |  | 140 | 1 (0.7) |  | 3 | 1 (33.3) |  |
|  | 2018 |  | 198 | 1 (0.5) |  | 4 | 1 (25.0) |  |
|  | 2019 |  | 280 | 7 (2.5) |  | 6 | 3 (50.0) |  |
|  | 2020 |  | 323 | 1 (0.3) |  | 7 | 1 (14.3) |  |
|  |  |  |  |  |  |  |  |  |
| Rewa | 2015 |  | 500 | 1 (0.2) |  | 19 | 1 (5.3) |  |
|  | 2016 |  | 675 | 58 (8.6) |  | 25 | 5 (20.0) |  |
| (46 Beef farms, | 2017 |  | 843 | 31 (3.7) |  | 29 | 5 (17.2) |  |
| 20 Dairy farms and | 2018 |  | 957 | 11 (1.1) |  | 30 | 5 (16.7) |  |
| 1 Holding Facility) | 2019 |  | 755 | 33 (4.4) |  | 31 | 7 (22.6) |  |
|  | 2020 |  | 564 | 27 (4.8) |  | 29 | 7 (24.1) |  |
|  |  |  |  |  |  |  |  |  |
| Serua | 2015 |  | 1330 | 17 (1.3) |  | 22 | 2 (9.1) |  |
|  | 2016 |  | 1454 | 111 (7.6) |  | 17 | 6 (35.3) |  |
| (98 Beef farms, | 2017 |  | 1506 | 16 (1.1) |  | 43 | 7 (16.3) |  |
| 21 Dairy farms and | 2018 |  | 1901 | 12 (0.6) |  | 64 | 6 (9.4) |  |
| 1 Holding Facility) | 2019 |  | 1675 | 66 (3.9) |  | 53 | 11 (20.8) |  |
|  | 2020 |  | 1382 | 47 (3.4) |  | 51 | 16 (31.4) |  |
|  |  |  |  |  |  |  |  |  |
| Tailevu | 2015 |  | 8865 | 649 (7.3) |  | 114 | 34 (29.8) |  |
|  | 2016 |  | 7131 | 791 (11.1) |  | 76 | 33 (43.4) |  |
| (191 Beef farms, | 2017 |  | 9591 | 518 (5.4) |  | 163 | 55 (33.7) |  |
| 139 Dairy farms and | 2018 |  | 8851 | 460 (5.2) |  | 128 | 39 (30.5) |  |
| 2 Holding Facilities) | 2019 |  | 9413 | 471 (5.0) |  | 171 | 70 (40.9) |  |
|  | 2020 |  | 10243 | 433 (4.2) |  | 202 | 75 (37.1) |  |

^1^ The number of unique identity farm operations is shown in parentheses.

^2^ Farms or animals tested or with positive results in that year; many farms and some animals were tested more than once per year; the same farm or animal can appear in more than one year, but within a year a farm or animal appears only once.

**Supplementary Table 3**: bTB test results in Western Division provinces between 2015 and 2020.

| **Western Division**  **Provinces^1^** | **Year** |  | **Animals tested^2^** | |  | **Farms tested^1^** | |
| --- | --- | --- | --- | --- | --- | --- | --- |
|  |  |  |  |  |  |  |  |
|  |  |  | **Total No.** | **No. positive (%)** |  | **Total No.** | **No. positive(%)** |
| Ba | 2015 |  | 641 | 3 (15.0) |  | 20 | 4 (0.6) |
|  | 2016 |  | 13 | - |  | 4 | - |
| (173 Beef farms, | 2017 |  | 278 | - |  | 25 | - |
| 90 Dairy farms and | 2018 |  | 1854 | 40 (2.2) |  | 105 | 21 (20.0) |
| 5 Holding Facilities) | 2019 |  | 1646 | 38 (2.3) |  | 110 | 23 (20.9) |
|  | 2020 |  | 1654 | 47 (2.8) |  | 67 | 15 (22.4) |
|  |  |  |  |  |  |  |  |
| Nadroga | 2015 |  | 106 | - |  | 4 | - |
|  | 2016 |  | 558 | - |  | 6 | - |
| (17 Beef farms, | 2017 |  | 670 | - |  | 11 | - |
| 5 Dairy farms and | 2018 |  | 46 | - |  | 3 | - |
| 3 Holding Facilities) | 2019 |  | 838 | 25 (3.0) |  | 10 | 4 (40) |
|  | 2020 |  | 805 | 16 (2.0) |  | 13 | 9 (69.2) |
|  |  |  |  |  |  |  |  |
| Navosa | 2015 |  | 476 | - |  | 5 | - |
|  | 2016 |  | 264 | - |  | 2 | - |
| (31 Beef farms, | 2017 |  | 517 | - |  | 6 | - |
| 3 Dairy farms and | 2018 |  | 257 | - |  | 5 | - |
| 3 Holding Facilities) | 2019 |  | 1031 | 11 (1.1) |  | 14 | 5 (35.7) |
|  | 2020 |  | 1252 | 15 (1.2) |  | 22 | 7 (31.8) |
|  |  |  |  |  |  |  |  |
| Ra | 2015 |  | 113 | - |  | 1 | - |
|  | 2016 |  | 78 | 3 (3.8) |  | 3 | 1 (33.3) |
| (19 Beef farms and | 2017 |  | 1257 | 20 (1.6) |  | 8 | 4 (50.0) |
| 1 Holding Facility) | 2018 |  | 496 | 1 (0.2) |  | 5 | 2 (40.0) |
|  | 2019 |  | 1693 | 23 (1.4) |  | 11 | 3 (27.3) |
|  | 2020 |  | 1100 | 5 (0.5) |  | 4 | 1 (25.0) |

^1^ The number of unique identity farm operations is shown in parentheses.

^2^ Farms or animals tested or with positive results in that year; many farms and some animals were tested more than once per year; the same farm or animal can appear in more than one year, but within a year a farm or animal appears only once.

**Supplementary Table 4:** bTB test results in Northern Division provinces between 2015 and 2020.

| **Northern Division**  **Provinces^1^** | **Year** |  | **Animal tested^1^** | |  | **Farms tested^1^** | |
| --- | --- | --- | --- | --- | --- | --- | --- |
|  |  |  |  |  |  |  |  |
|  |  |  | **Total No.** | **No. positive (%)** |  | **Total No.** | **No. positive (%)** |
| Bua | 2015 |  | - | - |  | - | - |
|  | 2016 |  | - | - |  | - | - |
| (12 Beef farms and | 2017 |  | 22 | - |  | 1 | - |
| 1 Holding Facility) | 2018 |  | - | - |  | - | - |
|  | 2019 |  | 23 | 2 (8.7) |  | 4 | 1 (25.0) |
|  | 2020 |  | 389 | 8 (2.1) |  | 9 | 4 (44.4) |
|  |  |  |  |  |  |  |  |
| Cakaudrove | 2015 |  | - | - |  | - | - |
|  | 2016 |  | - | - |  | - | - |
| (21 Beef farms and | 2017 |  | 216 | - |  | 2 | - |
| 1 Holding Facility) | 2018 |  | 1659 | 11 (0.7) |  | 9 | 3 (33.3) |
|  | 2019 |  | 2215 | 97 (4.4) |  | 5 | 3 (60.0) |
|  | 2020 |  | 2878 | 94 (3.3) |  | 20 | 8 (40.0) |
|  |  |  |  |  |  |  |  |
| Macuata | 2015 |  | - | - |  | - | - |
|  | 2016 |  | - | - |  | - | - |
| (12 Beef farms and | 2017 |  | 16 | - |  | 3 | - |
| 3 Holding Facility) | 2018 |  | 115 | 1 (0.9) |  | 4 | 1 (25.0) |
|  | 2019 |  | 31 | - |  | 3 | - |
|  | 2020 |  | 379 | 12 (3.2) |  | 11 | 5 (45.5) |

^1^ The number of unique identity farm operations is shown in parentheses

^2^ Farms or animals tested or with positive results in that year; many farms and some animals were tested more than once per year; the same farm or animal can appear in more than one year, but within a year a farm or animal appears only once.

**Supplementary Table 5:** Missing animals on dairy farms from Central Division by size of operation reported in *Bovibase* in 2019 and 2020.

| **Operation size** | **Year** |  | **Animals^a^** | | |  | **Farms** | | |
| --- | --- | --- | --- | --- | --- | --- | --- | --- | --- |
|  |  |  | **No. tested** | **No. missing** | **% missing** |  | **No. tested** | **No. with missing animals** | **% with missing animals** |
| Commercial | 2019 |  | 7,820 | 1192 | 15.2 |  | 82 | 50 | 61.0 |
| ≥ 41 cattle | 2020 |  | 8,314 | 2266 | 27.3 |  | 83 | 83 | 100.0 |
|  |  |  |  |  |  |  |  |  |  |
| Semi-commercial | 2019 |  | 2934 | 401 | 13.7 |  | 111 | 44 | 39.6 |
| ≥ 16 - ≤40 cattle | 2020 |  | 2969 | 1390 | 46.8 |  | 117 | 113 | 96.6 |
|  |  |  |  |  |  |  |  |  |  |
| Subsistence | 2019 |  | 491 | 65 | 13.3 |  | 47 | 18 | 38.3 |
| ≥ 1 – ≤15 cattle | 2020 |  | 746 | 269 | 36.1 |  | 55 | 50 | 90.9 |

^a^ The median values per BTEC testing visit during each year.

**Supplementary Table 6:**  Outcome of culture of tissues from bTB reactors sent to Fiji Veterinary Pathology Laboratory from 2018 to 2020

|  |  |  |  | |  | | |  |  | | | |  |
| --- | --- | --- | --- | --- | --- | --- | --- | --- | --- | --- | --- | --- | --- |
| **Farm type** |  | **Year** |  | **bTB reactor animals** | | |  | **Visible lesions** | |  | **No visible lesions** | | |
|  |  |  |  | **No. examined** | | **No. culture-positive (%)** |  | **No. examined** | **No. culture-positive (%)** |  | **No. examined** | **No. culture-positive (%)** | |
| All |  | 2018 |  | 165 | | 110 (66.7) |  | 23 | 16 (70.0) |  | 142 | 94 (66.2) | |
|  |  | 2019 |  | 512 | | 463 (90.0) |  | - | - |  | 512 | 463 (90.0) | |
|  |  | 2020 |  | 520 | | 354 (68.0) |  | - | - |  | 520 | 354 (68.0) | |
